# Myocardin regulates exon usage in smooth muscle cells through induction of splicing regulatory factors

**DOI:** 10.1007/s00018-022-04497-7

**Published:** 2022-08-01

**Authors:** Li Liu, Dmytro Kryvokhyzha, Catarina Rippe, Aishwarya Jacob, Andrea Borreguero-Muñoz, Karin G. Stenkula, Ola Hansson, Christopher W. J. Smith, Steven A. Fisher, Karl Swärd

**Affiliations:** 1 Department of Experimental Medical Science, BMC D12, SE-22184 Lund, Sweden; 2 Department of Urology, Qingyuan People’s Hospital, The Sixth Affiliated Hospital of Guangzhou Medical University, Qingyuan, China; 3 Department of Clinical Sciences, Lund University Diabetes Centre, CRC, 21428 Malmö, Sweden; 4 Department of Biochemistry, University of Cambridge, Cambridge, UK; 5 Institute for Molecular Medicine Finland (FIMM), Helsinki University, Helsinki, Finland; 6 Department of Medicine (Cardiology) and Physiology and Biophysics, University of Maryland-Baltimore, Baltimore, MD 21201 USA

**Keywords:** Splicing code, Actin-binding, Focal adhesion, Heart, Differentiation

## Abstract

Differentiation of smooth muscle cells (SMCs) depends on serum response factor (SRF) and its co-activator myocardin (*MYOCD*). The role of MYOCD for the SMC program of gene transcription is well established. In contrast, the role of MYOCD in control of SMC-specific alternative exon usage, including exon splicing, has not been explored. In the current work we identified four splicing factors (*MBNL1*, *RBPMS*, *RBPMS2*, and *RBFOX2*) that correlate with *MYOCD* across human SMC tissues. Forced expression of MYOCD family members in human coronary artery SMCs in vitro upregulated expression of these splicing factors. For global profiling of transcript diversity, we performed RNA-sequencing after MYOCD transduction. We analyzed alternative transcripts with three different methods. Exon-based analysis identified 1637 features with differential exon usage. For example, usage of 3´ exons in *MYLK* that encode telokin increased relative to 5´ exons, as did the 17 kDa telokin to 130 kDa MYLK protein ratio. Dedicated event-based analysis identified 239 MYOCD-driven splicing events. Events involving *MBNL1*, *MCAM*, and *ACTN1* were among the most prominent, and this was confirmed using variant-specific PCR analyses. In support of a role for RBPMS and RBFOX2 in MYOCD-driven splicing we found enrichment of their binding motifs around differentially spliced exons. Moreover, knockdown of either RBPMS or RBFOX2 antagonized splicing events stimulated by MYOCD, including those involving *ACTN1*, *VCL*, and *MBNL1*. Supporting an in vivo role of MYOCD-SRF-driven splicing, we demonstrate altered *Rbpms* expression and splicing in inducible and SMC-specific Srf knockout mice. We conclude that MYOCD-SRF, in part via RBPMS and RBFOX2, induce a program of differential exon usage and alternative splicing as part of the broader program of SMC differentiation.

**Supplementary Information:**

The online version contains supplementary material available at 10.1007/s00018-022-04497-7.

## Introduction

Smooth muscle cells (SMCs) are not terminally differentiated and can change their phenotype in response to environmental cues. This allows SMCs to switch from a contractile and highly differentiated phenotype to several less differentiated phenotypes [1], one of which is called synthetic. These transitions, referred to as phenotypic modulation, play a central role in numerous vascular diseases. Critical for the contractile phenotype is the powerful transcriptional co-activator myocardin (*MYOCD*) [2–4], which associates with serum response factor (*SRF*) on genomic elements called CArG boxes to control a gene program required for contractile differentiation and SMC identity [5]. Inducible disruption of MYOCD-SRF activity in SMCs of adult mice circumvents developmental lethality and leads to impaired contractility and severe morbidities involving numerous organ systems, including blood vessels, the gastrointestinal tract, and the urogenital tract [6–8].

Pre-messenger RNAs (pre-mRNAs) derived from multi-exon genes are subject to alternative splicing (AS), and this increases transcriptomic and proteomic diversity several-fold [9–11]. On average, each gene has 6.3 alternatively spliced transcripts of which 3.9 are protein-coding [12]. An important theme in AS is the presence of conserved AS signatures that differ between tissues [13, 14]. Such signatures are likely driven in part by tissue-specific RNA-binding splicing factors [10, 11, 15], but the identity and combinations of splicing regulators specific to individual cell types have remained elusive [16]. Transcriptomic diversity is further increased by alternative internal promoters and transcription start sites, and such events may be more common than AS [17]. That AS is widespread and plays critical roles in human disease is clear [18, 19], but the magnitude of its proteomic impact has been questioned [20]. This suggests that studies aiming to assess tissue specific AS should also aim to probe its proteomic consequences.

Beyond high expression of differentiation markers, certain AS events define contractile SMCs [15, 21, 22]. For example, smooth muscle myosin, encoded by *MYH11*, has different splice variants [23] that influence shortening velocity and force development [24]. Tropomyosin 1 (*TPM1*), similarly, is spliced in an SMC-specific manner [25, 26], as is caldesmon (*CALD1*) [27]. Yet another example is the L-type Ca^2+^-channel [28], for which SMC-specific AS determines sensitivity to channel blockers of the dihydropyridine class, a group of medicines used to reduce blood pressure [29, 30]. Developmentally regulated splicing of the myosin phosphatase targeting subunit (MYPT1 or *PPP1R12A*) [31], that influences nitric oxide-dependent arterial dilatation and blood pressure has also been uncovered [32]. *MYOCD* itself is regulated by AS, with an N-terminally truncated SMC isoform that preferentially interacts with SRF [33, 34]. The existence of an SMC-specific AS code supports the presence of SMC-enriched splicing factors. Indeed, a recent study indicated that Rbpms (RNA-binding protein with multiple splicing) may be one such factor [35], but several others have been shown to influence AS in SMCs [15], including PTBP1 [22], MBNL1 [26], TRA2B [36], RBFOX2 [37], and QKI [38].

In the current work we aimed to define splicing factors enriched in SMCs by leveraging RNA-sequencing data in correlation analyses, to explore if the identified splicing factors are regulated by MYOCD, and to determine whether MYOCD influences transcript isoform diversity. We find that MYOCD regulates the splicing factors RBPMS and RBFOX2 at the mRNA and protein levels in human coronary artery SMCs. Using RNA-sequencing and three different methods to assess transcript diversity, we define 1637 features of differential exon usage, 239 splicing events, and 515 genes with isoform switches, of which 151 have predicted functional consequences. Moreover, we find that binding sites for RBPMS and RBFOX2 are enriched around exons targeted by MYOCD, and that knockdown of either RBPMS or RBFOX2 mitigates the effect of MYOCD on splicing. MYOCD therefore contributes to SMC-specific transcript diversity by regulating the splicing factors RBPMS and RBFOX2, but also by favoring alternative promoters and 3´ ends.

## Materials and methods

### RNA correlation analyses

RNA-sequencing data from different human organs was downloaded from the GTExPortal.org in mid-2020, and the overall RNA-level of *MYOCD* (in transcripts per million, TPM) was correlated to all other transcripts in individual organs using the Pearson method and Excel. For the current analyses we used the 26 tissues with highest median *MYOCD* expression (from aorta to skeletal muscle), with a minimum, maximum, and median of 9, 803, and 390 individuals per tissue. The sum of Pearson correlation coefficients was calculated for each transcript across tissues yielding an Rsum value that was sorted in descending order. *MYOCD* correlations versus splicing factors from the top percentile of Rsum values were subsequently tested using the Spearman method in GraphPad Prism.

To support a role of MYOCD-driven splicing in human tissues, we examined key splicing findings using transcript, exon and junction data for aorta, tibial artery, coronary artery, and esophagus muscularis in GTExPortal. Pairwise Spearman’s correlations and the Dunn and Clark test [39] for comparing dependent overlapping correlations were used to test if our key alternative splicing findings were differently correlated with overall expression of MYOCD.

### Cell culture and viral overexpression

Cryopreserved human coronary artery SMCs were purchased from Thermo Scientific/Gibco (C-017-5C). They were cultured using either Human Vascular Smooth Muscle Cell Basal Medium (Life Technologies, M231500) with addition of growth supplement (SMGS, Life Technologies, S00725) and PEST (50U/50 μg/ml, Biochrom, A2212) or Smooth Muscle Cell Growth Medium (Sigma-Aldrich, #311–500) with addition of PEST in a standard cell culture incubator (37 °C, 95% air, and 5% CO_2_). Smooth muscle cells from the human bladder were obtained in a prior study [40] and cryopreserved. After thawing, cells were cultivated in DMEM/Ham’s F-12 medium (Biochrom, FG4815) containing 10% fetal bovine serum (Biochrom, S0115) and PEST. All cells were used in passages 3–8. Adenoviruses were purchased from Vector Biolabs: Ad-h-MRTFA/eGFP (ADV-215499), Ad-h-MRTFB (ADV-215500), Ad-h-MYOCD (ADV-216227), Ad-CMV-Null (#1300), Ad-h-shSRF (shADV-224323), and Ad-GFP-U6-shRNA (#1122). Transductions were done at the indicated concentrations (multiplicities of infection, MOI). As negative controls for overexpression and knockdown we used Ad-CMV-Null and Ad-GFP-U6-shRNA, respectively. Transduced cells were harvested at 96 h, at144h, or at 192 h as indicated. The viral vector used for MYOCD encodes a transcript without exon 2a, expressed primarily in heart, but to some extent (≈ 25%), in SMCs.

### RT-qPCR

Cells were washed (PBS, P4417, Sigma-Aldrich) and then lysed in RLT Lysis buffer from the RNeasy mini kit. The RNeasy mini kit (Qiagen #74106) and a QIAcube workstation were used for RNA isolation. Purity and concentration of isolated RNA was determined using a NanoDrop 2000c instrument (Thermo Scientific). *MRTF-A* primer (Forward: 5´-ATGCCGCCTTTGAAAAGTCCA-3´, Reverse: 5´-TCTTCCGTTTGAGATAGTCCTCT-3´) was ordered from Eurofins Genomics. This primer and QuanTitect primer assays for *RBPMS* (QT00042679), *RBPMS2* (QT01034824), *MBNL1* (QT00077952), *RBFOX2* (QT00039907), *MYOCD* (QT00072884), *MRTF-B* (QT00010115), *MYH11* (QT00069391), *MCAM* (QT00079842), *KCNMB1* (QT00080493), *CAV1* (QT00012607), *SRF* (QT00084063), *ACTA2* (QT00088102), and *18S* (QT00199367), along with the QuantiFast SYBR Green RT-PCR kit (Qiagen, #204,156) were used to prepare mixes (10 µl) that were run using the StepOnePlus thermal cycler (Applied Biosystems). Exact primer sequences are not revealed by Qiagen. 18S was used as a reference gene when calculating fold changes (FC) using the Pfaffl method.

### Differential gene expression and alternative splicing analyses

A detailed account of the RNA-sequencing experiment along with processed data was published elsewhere [41]. Raw bulk RNA-Seq data is available at the Sequence Read Archive with the BioProject PRJNA731342. Briefly, sequencing was done at the local core facility using a NextSeq 500/550 High Output Kit v2.5 and the Illumina NextSeq 500 instrument. The average sequencing depth was 52 (42–57) million 75 bp paired-end reads per sample. Reads were mapped with STAR 2.7.6 [42] in 2-pass mode and counted with featureCounts 2.0.1 [43]. Differential gene expression analysis was done using DESeq2 1.26.0 [44].

We combined three main strategies to assess splicing: exon-based, event-based, and isoform-based analysis [45]. (i) The exon-based analysis was performed with DEXSeq 1.32.0 [46] that uses exon counts to test for differential exon usage between conditions. This method covers most genes and discovers cases of potential alternative splicing with high sensitivity. However, inferences regarding the type of splicing event or consequences for specific transcript isoforms are precluded, and thus predictions regarding biological consequences are difficult. For functional inferences of the DEXSeq results, we used WebGestaltR [47] to run enrichment analysis with different functional databases using the genes showing differential exon usage. (ii) We used rMATS 4.1.1 for the event-based analysis [48]. This analysis identifies splicing events supported by overlapped sequencing reads, and thus provides grounds for biological inferences. Events detected by rMATS can often be validated by PCR, but rather few events are detected due to the limited coverage. (iii) Isoform-based splicing analysis was done with IsoformSwitchAnalyzeR 1.8.0 [49]. It is the most informative analysis of those used in terms of functional inferences, as knowledge of isoform switches between conditions allows assessing functional consequences based on the prediction of Open Reading Frames (ORF), protein domains (via Pfam), signal peptides (via SignalP), intrinsically disordered regions (IDR, via IUPred2A), coding potential (via CPAT), and sensitivity to Non-sense Mediated Decay (NMD). The drawback of the isoform-based approach is the accuracy of read assignment to different isoforms that originate from the same gene as such isoforms are similar in their sequences. We used Salmon 1.3.0 for transcripts/isoforms counts which performs reasonably well in this task [50]. The code and conda environment for the differential gene expression and alternative splicing analyses are available at https://github.com/LUDC-bioinformatics/SMC_MYOCD_splicing.

### Western blotting

Cells were thoroughly washed in ice cold PBS followed by addition of lysis buffer (70 μl 60 mM Tris–HCl, 2% SDS, 10% glycerol, pH 6.8), and scraping using a rubber policeman. Protein concentration was analyzed using the BIO-RAD DC protein assay kit (#500–0112). Mercaptoethanol (to 5%) and bromophenol blue were added, and the protein concentration was adjusted to 1 μg/μl. After heating (95 °C, 5 min), samples were stored at − 80 °C. For one-dimensional protein separation we used pre-cast gels (BIO-RAD, #5,671,084 and #5,671,085) that were run in an electrophoresis unit using Tris/Glycine/SDS buffer (BIO-RAD, #161–0732) until the front ran off. The Trans-Blot Turbo system was used for protein transfer to 0.2 μM nitrocellulose (BIO-RAD, #170–4159), and membranes were blocked for 2 h in Casein block (BIO-RAD, #161–0782) at room temperature. Antibodies were added directly to the blocking buffer at recommended dilutions. The following primary targets were assayed: RBPMS (Millipore Sigma, HPA056999), RBFOX2 (Sigma-Aldrich, HPA006240), MBNL1 (Abcam Biochemicals, ab108519), MYLK (Abcam, ab76092), HSP90 (BD Biosciences, 610,418), SLMAP (Millipore Sigma, HPA002357), ACTN1 (Sigma-Aldrich, HPA006035), FLNA (Abcam, ab76289), TPM1 (Cell Signaling Technology, 3910S), CALD1 (Cell Signaling Technology, 12503S), VCL (Cell Signaling Technology, 13901S), SORBS1 (Abcam, ab224129), p-MLC2 (Cell Signaling Technology, 3675S), MLC2 (official gene symbol is *MYL9*, Cell Signaling Technology, 3672S), and GAPDH (EMD Millipore, MAB374). The MYLK antibody was raised using a peptide corresponding to amino acids 1850–1950 in the C terminus. It is therefore predicted to detect telokin in addition to long MYLK isoforms. Membranes were enclosed in bags with a small volume of diluted antibody and maintained at 4 °C with tumbling or shaking for 48 h. After three 10 min washes in Tris-buffered saline (BIO-RAD, 170–6435) with 0.1% Tween (BIO-RAD, 161–0781), membranes were incubated with secondary HRP-conjugated antibodies (1:10,000, Cell Signaling Technology #7074S and #7076S) for 2 h. After three final washes, signals were developed using West Femto substrate (Thermo Fisher Scientific, #34,096) in an Odyssey Fc Imager (LI-COR Biosciences). Blots were stripped (Thermo Scientific, 46,430) for 30 min, at 60 °C, and recycled for detection of a house-keeping protein.

### Immunofluorescence

Cells were fixed in 4% paraformaldehyde in Krebs–Ringer Bicarbonate buffer for 30 min at room temperature. To unmask antigen-binding sites, cells were incubated in 0,05% antigen retrieval solution (0,5% trypsin, 1% (*w*/*v*) CaCl, dH20, pH 7.8) for 15 min in a humidified container at 37 °C. Immediately after removing the trypsin solution, cells were blocked and permeabilized in blocking buffer (1% (*w*/*v*) bovine serum albumin, 1% goat serum, 1% (v/v) Triton) for 30 min at room temperature. Cells were labeled with rabbit-MYLK (Abcam, ab76092) in blocking buffer without Triton at 1:200 dilution overnight at 4 °C. The next day, cells were washed 2 times and labeled with secondary antibody (Alexa Fluor-488, Invitrogen, A-11034) at room temperature for 1 h. Hoechst (Invitrogen, H3569) and Phalloidin (Invitrogen, A22287) were added to visualize nuclei and actin filaments, respectively. Images were acquired using a Nikon A1 plus confocal microscope using 20 × magnification, NA 1.40 (Nikon Instruments Inc.). A z-stack was acquired using 8 averaging and a 17.88 pinhole size. Image projections and mean intensity values of the MYLK signal were made and calculated using the Fiji software.

### Isoform-specific PCR analyses

RNA was extracted from transduced cells (null and MYOCD), and cDNA was generated using the High-Capacity cDNA Reverse Transcription Kit (Thermo Fisher, 4,368,814). After dilution, reactions were run for 35 cycles (denaturation: 94 °C, 30 s; annealing: 61 °C, 30 s; extension: 72 °C, 1 min) using PCR. Specific primers were obtained from VastDB (http://vastdb.crg.eu/wiki/Main_Page) and produced by Eurofins Genomics. Amplicons were separated for 2.5 h-4 h on 2% agarose gels containing the nucleic acid stain Gelred (VWR, 41,003), and images were captured using an Odyssey Fc Imager (LI-COR Biosciences). For quantitative analysis, we divided the intensity of the band of interest (typically the weakest) with all bands in the same lane (× 100) and refer to this as percent spliced in (PSI, varying from 0 to 100). One exception is panel R in the figure presenting the effects of RBPMS and RBFOX2 knockdown, and where human bladder SMCs were used. In these cells, and contrasting to coronary SMCs, the major MBNL1 band was the lowest one, but we still used it in the numerator as we wanted the analysis to be comparable to data in panel I in the same figure.

### Enrichment of binding motifs for splicing factors

Motif enrichment analyses were performed using MATT [51] essentially as described [35]. Briefly, exons in transcripts identified by rMATS (FDR < 0.05 and |ΔPSI|> 5%, PSI = percent spliced in) were used to test enrichment or depletion of recognition elements for RBPMS (CACn(1–12)CAC), RBFOX (GCAC/TG), and MBNL (C/TGCC/T). RNA maps were obtained using the rna_maps command, and as a background, an unregulated set of 2000 exons was used.

### RNA silencing

*RBPMS* siRNA (si-RBPMS, SR307544) and *RBFOX2* siRNA (si-RBFOX2, SR308351) were purchased from OriGene. Cells were transduced with Null or MYOCD viruses (200MOI) and 50 nM si-RBPMS or si-RBFOX2 was transfected into cells after 48 h using siTran 2.0 siRNA transfection reagent (OriGene, TT320001) according to the manufacturer’s instructions. Cells were harvested for RNA isolation at 96 h.

### Depolymerization of actin

To depolymerize actin, cells were treated with Latrunculin B (LatB100 nM, Calbiochem, 428,020) or vehicle (DMSO) in serum-free medium for 20 h. Cells were then allowed to recover for 4 h in serum-containing medium without LatB. At 24 h LatB was again added. After 4 cycles, cells were harvested, and RNA was extracted.

### Inducible smooth muscle knockout of Srf

Srf-floxed mice (Srf^fl/fl^) were bred with hemizygous Myh11-Cre/ER^T2^ mice as previously described [41]. Recombination was induced by intraperitoneal injection of tamoxifen (1 mg/mouse/day) for 5 consecutive days in 8-12w old mice. These mice are referred to as knockouts (KO). Control mice were either Cre-positive Srf^fl/fl^ mice injected with ethanol/sunflower oil (1:10) (vehicle controls), or Cre-negative Srf^fl/fl^ mice injected with tamoxifen (tamoxifen controls), in an identical manner. There were no differences between the two control groups in the analyses, so the two control groups were pooled and simply referred to as wild type (WT). Mice were euthanized by cervical dislocation 21-25d post-injections, and the aorta and bladder were excised. After fine dissection, tissues were either fixed and imaged [52], snap frozen for biochemical experiments, or mounted in myographs [41, 53] for force measurements. Mouse RT-qPCR primers were from Qiagen (*Srf*: QT00126378, *Myh11*: QT02327626, *Cnn1*: QT00105420, *Tagln*: QT00165179, *Rbpms_vb*: QT01553972, *Rbfox2*: QT00146216, and *18 s*: QT02448075). Primers for detection of Myocd-Ex2a were obtained from the literature [33], and other primers for isoform-specific PCR analyses were from VastDB. All of them were obtained from Eurofins Genomics. For western blotting and imaging, we used antibodies against Myh11 (Abcam, ab53219), Srf (Cell Signaling Technology, 5147S; Proteintech, 16,821–1-AP), Cnn1 (Abcam, ab46794), and Tagln (Sm22, Abcam, ab14106). The remainder of the antibodies used in mice were the same as those used in human cells.

### Statistics

Normality of data was tested using Shapiro–Wilk’s test, and the *F*-test or Brown-Forsythe test was used for checking homogeneity of variances. For two-group comparisons, 2-sided student’s *t*-test for unpaired comparisons was used in cases of normally distributed and equal variance data. Welch’s *t* test was used if the normality criterion was met but variances were inhomogeneous. If the normality criterion was not met, we used Mann Whitney *U*. For multi-group comparisons we used ordinary one-way ANOVA followed by Tukey’s or Dunnett’s post-hoc tests in cases of normality and homogenous variances. If the normality test failed, Kruskal–Wallis one-way ANOVA with Dunn's post hoc test was used. If the normality test passed but the homogeneity test failed, Welch’s one-way ANOVA test with Dunnett's T3 post hoc test was performed. The Spearman method was used to test individual correlations. *P* < 0.05 was considered significant. All statistical tests were performed in GraphPad Prism version 9.2.0.

## Results

### RBPMS and RBFOX2 correlate with MYOCD across human smooth muscles

**Fig. 1:**
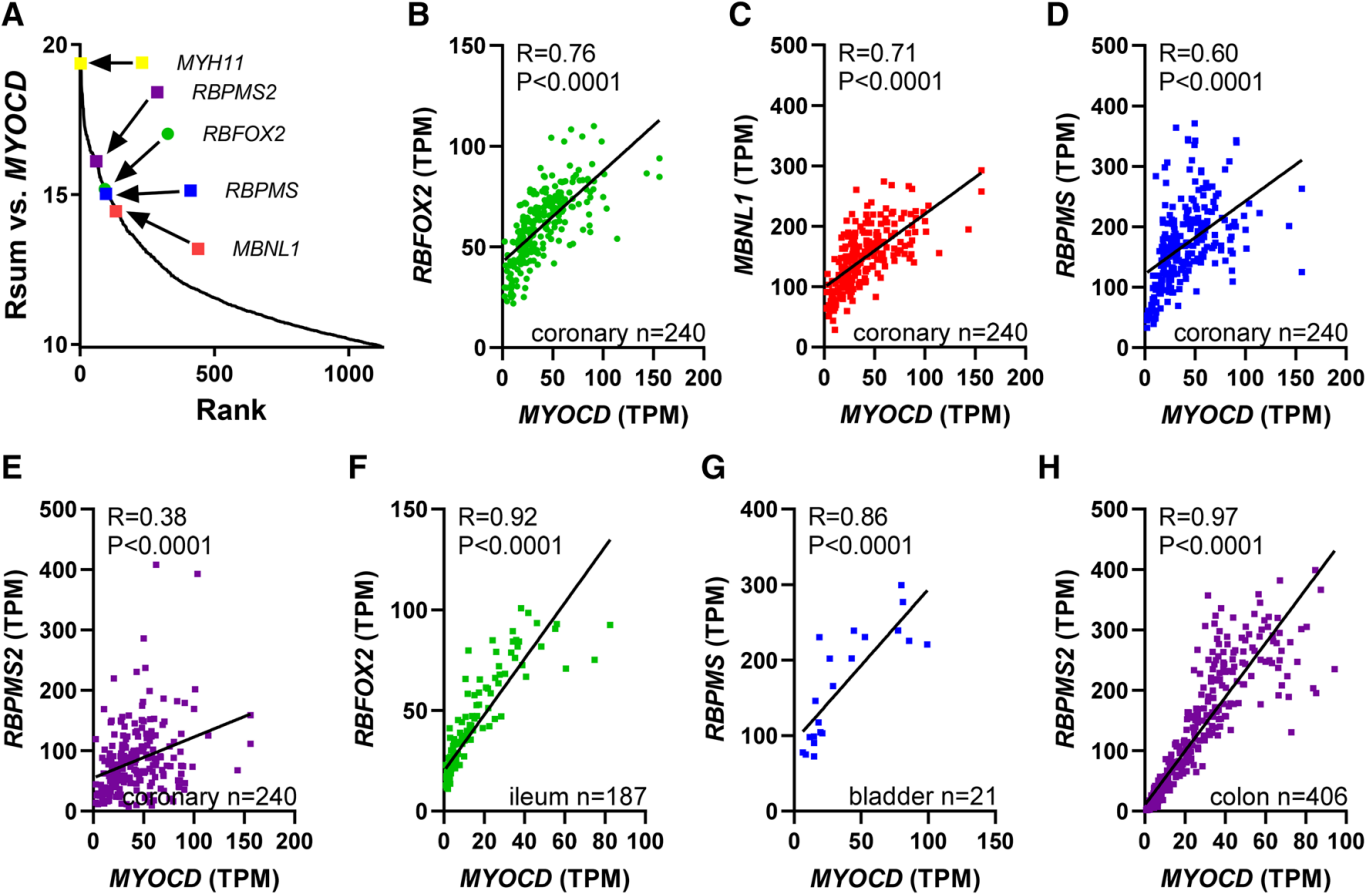
Correlation analyses suggest that myocardin may target splicing regulatory proteins to effectuate changes in splicing. RNA-sequencing data from the GTExPortal.org was used to correlate myocardin (*MYOCD*), a master regulator of smooth muscle cell (SMC) differentiation, with all other transcripts in 26 tissues. Four splicing factors were found in the positive extreme (top 1%) of the sum of correlation coefficients across tissues (Rsum, **A**). **B**–**E** Examples of correlations between myocardin (MYOCD) and the four splicing factors in the coronary artery. These panels also show the R and P-values (Spearman), and illustrate the size (*n*-values) for some of the datasets underlying the analysis in A. **F**–**H** Additional examples from the analysis in A. These findings suggested the possibility that myocardin, and perhaps all myocardin related transcription factors (MRTFs), may target splicing regulatory proteins, thus contributing to the splicing code in SMCs

Using RNA-sequencing data downloaded from the GTExPortal.org, we noted that a handful of splicing factors were among transcripts that are highly correlated (i.e., they were within the top percentile of Rsum-values) with myocardin (*MYOCD*) across 26 human tissues containing SMCs (Fig. 1A, *n* = 9 to *n* = 803). The splicing factors correlating best with *MYOCD* were *RBPMS2*, *RBFOX2*, *RBPMS*, and *MBNL1* (colored symbols in Fig. 1A), each of which has previously been implicated in SMC AS regulation. Individual correlations versus *MYOCD* are shown for the coronary artery in Fig. 1B–E, and three additional examples from the overall analysis in Fig. 1A are shown in Fig. 1F–H. *MYOCD* correlated positively with *RBPMS* in all the three arteries represented in the database, and indeed across different SMC tissues, arguing that this association is shared among arteries independently of anatomical location. *MRTFB*, a co-activator that is closely related to *MYOCD*, correlated less well to RBPMS across tissues. We have previously seen that *MYOCD* correlates at the mRNA level with many of the transcripts that it regulates [41, 52, 54]. We therefore hypothesized that MYOCD regulates the expression of these RNA splicing proteins to cause SMC-specific splicing of pre-mRNAs. We also reasoned that MYOCD may affect transcript diversity by more direct means, by favoring e.g., alternative transcription start sites and 3´ ends.

### Forced expression of MYOCD increases RBPMS and RBFOX2 at the mRNA and protein levels

**Fig. 2:**
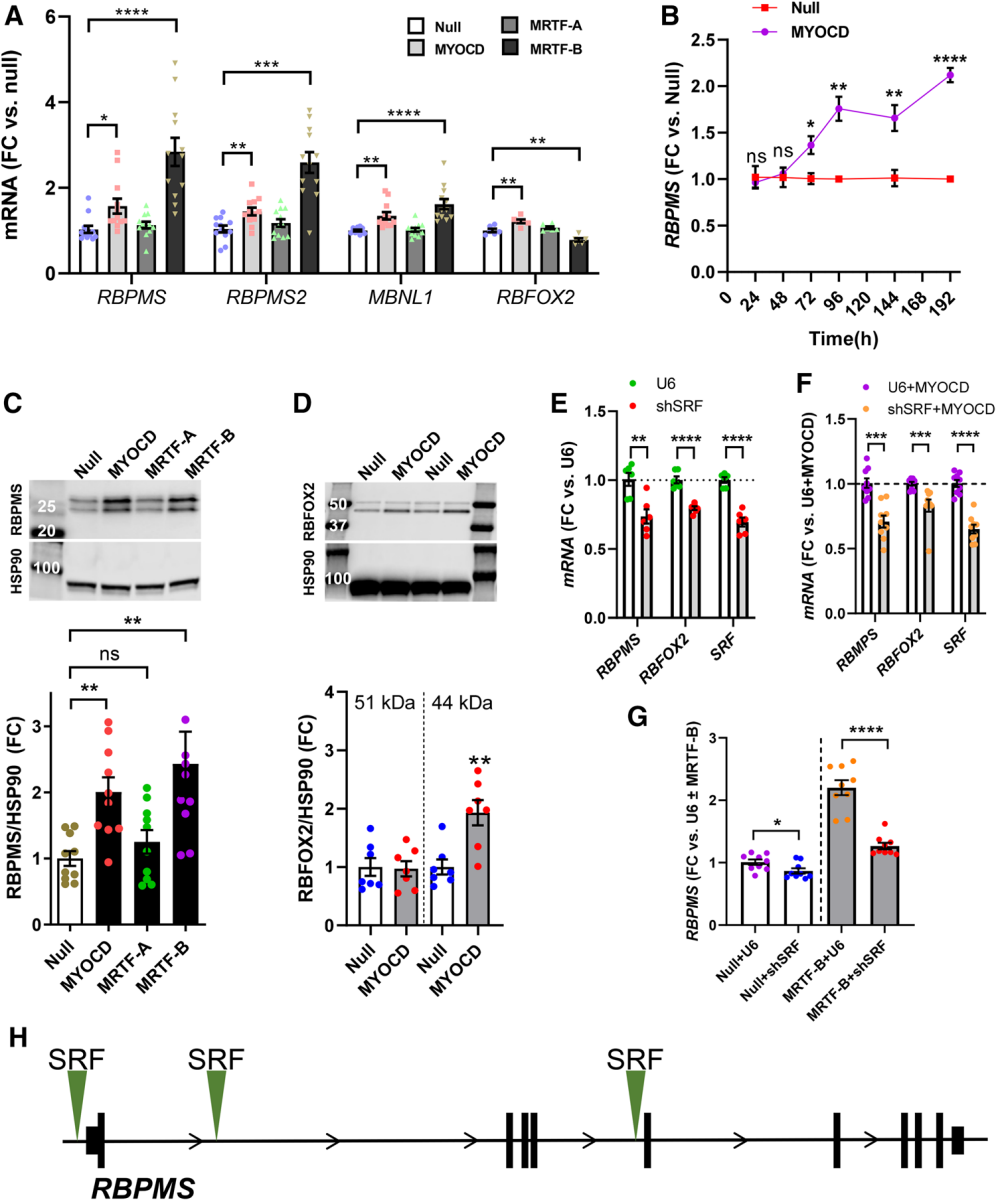
MYOCD and MRTF-B affect expression of splicing factors. To approach the hypothesis that MYOCD targets splicing factors, we assayed the identified splicing factors at the mRNA level following adenoviral overexpression of myocardin (MYOCD) and the two myocardin family members MRTF-A and MRTF-B. Adenoviruses (200 MOI) were added to human coronary artery SMCs in culture and cells were harvested at 4 days. Transcript levels were determined by RT-qPCR and mRNA fold changes (FC) are shown in (**A**) (*n* = 6–12 throughout, error bars represent SEM). **B** A time-course experiment where Ad-h-MYOCD or Ad-null viruses were added at 0 h and cells were harvested at different times, followed by RT-qPCR for RBPMS (*n* = 4 null and 4 MYOCD at each time). Panels C and D show western blots for RBPMS (6 days, to account for any delay between mRNA and protein) and RBFOX2 (8 days), respectively. Compiled western blot data is shown below the membranes (*n* = 10 for **C** and 6–7 for **D**). HSP90 was used to ascertain equal protein loading. MYOCD binds to DNA via serum response factor (SRF). To examine the role of SRF, a short hairpin (shSRF) virus was used for knockdown. *RBPMS* and *RBFOX2* were then assayed using RT-qPCR (panel E, coronary artery SMCs under basal conditions, and (**F**), bladder SMCs transduced with MYOCD, *n* = 6 throughout). SRF is the positive control. The control construct in this case is referred to as U6 or U6 + MYOCD. SRF knockdown was also combined with MRTF-B overexpression (**G**), showing that the RBPMS increase with MRTF-B depended on SRF (*n* = 9). In (**E**, **F**), cells were transduced with virus for 8 days, and in (**G**), cells were transduced for 6 days. Panel H shows the RBPMS gene locus with ChIP-seq data for SRF (green triangles, from the USCS genome browser)

To directly test if MYOCD regulates the expression of splicing factors, we overexpressed MYOCD in human coronary artery SMCs using an adenoviral vector. We chose overexpression over silencing because MYOCD levels fall substantially when SMCs are propagated in cell culture [3]. Moreover, some MYOCD target genes, while universally active in SMCs in tissue, are silent in cultured coronary artery SMCs [55]. The effect of MYOCD was compared with family members MRTF-A and MRTF-B. Null virus was used as control. Forced expression of MYOCD (giving 20–40% positive nuclei) increased *RBPMS*, *RBPMS2*, *MBNL1*, and *RBFOX2* mRNAs (Fig. 2A, pink squares vs. blue circles). No effect was seen with MRTF-A (Fig. 2A, green triangles). MRTF-B (beige triangles) had the same effect as MYOCD on the abundance of all transcripts assayed except for *RBFOX2* (ANOVA-Dunnett). Overexpression of MRTF-B did not increase the level of MYOCD or vice versa (Online resource 1A-C), rather, there was some degree of mutual antagonism. Time-course studies demonstrated a time-dependent increase of *RBPMS* following MYOCD transduction (Fig. 2B). In addition, MYOCD and MRTF-B increased RBPMS at the protein level when assayed at 6d, a time chosen to account for any delay between mRNA and protein (Fig. 2C). No significant effect was seen with MRTF-A (Fig. 2C). Protein levels, determined by western blotting, of MBNL1, RBPMS2, and RBFOX2 did not change after forced expression of MYOCD at 6d (data not shown). However, at 8d of MYOCD transduction, RBFOX2 increased (Fig. 2D) while MBNL1 remained unchanged (not shown). MYOCD thus increases RBPMS and, with a delay, RBFOX2 at the protein level.

To examine involvement of SRF we next used a short hairpin construct (Ad-shSRF) for silencing. The *RBPMS* and *RBFOX2* mRNA levels were reduced by SRF silencing under basal conditions (Fig. 2E) in human coronary artery SMCs. Moreover, the mRNA level of *RBPMS* and *RBFOX2* were decreased by SRF silencing in MYOCD-transduced human bladder SMCs (Fig. 2F). Similarly, the increase of *RBPMS* caused by MRTF-B was antagonized by SRF silencing in human coronary artery SMCs (Fig. 2G). ENCODE ChIP-sequencing data in the Genome Browser shows binding of SRF in the *RBPMS* promoter and in two introns (Fig. 2H, green triangles). SRF binding in the *RBPMS* promoter was located ≈ 2 kb upstream of the transcription start site of the longest transcript and overlapped the CArG-like sequence CCTTAACTGG. Only one SRF binding site was present at the *RBFOX2* locus, and it was distant from the transcription start site (≈ 50 kb upstream). Taken together, these findings suggest SRF-dependent expression of *RBPMS* and *RBFOX2*.

### RNA-sequencing after overexpression of MYOCD confirms altered expression of the splicing factors *RBPMS* and *RBFOX2*

Next, to examine the impact of MYOCD on transcript diversity, we designed an RNA-sequencing (RNA-seq) experiment comparing MYOCD and null transduced cells (Online resource 2A). Following quantification of four known target transcripts by RT-qPCR (Online resource 2B), four samples from each group were chosen for sequencing. As previously reported [41], over-expression of MYOCD has a profound effect on total gene expression causing differential expression of 11,363 genes (FDR < 0.01, Online resource 2C), including the well-known MYOCD targets *MYH11*, *ACTA2*, and *CNN1*.

Focusing on the differential expression analysis, we examined the fate of the four splicing factors. *MBNL1*, *RBFOX2*, and *RBPMS* were increased by MYOCD (Online resource 2D). *RBPMS2*, however, was unchanged (Online resource 2D). We also manually surveyed RNA-binding proteins using a cut-off for significance (FDR < 10^–5^) and identified 18 splicing factors that changed. *MBNL1* was the most increased, and *RNPC3* was the most reduced (Online resource 2E). *QKI*, previously found to be upregulated on modulation of SMCs towards the synthetic phenotype [34], was reduced (Online resource 2E). Two of the splicing factors identified in the RNA-seq experiment also ranked highly in the analysis in Fig. 1A. These were RBM24 (rank 486, i.e., top 1%), and STAU1 (rank 1005). We also performed Venn analyses using (i) a list of 1542 RNA-binding proteins [56], (ii) the top 1% of transcripts in the MYOCD correlation analysis, and (iii) all transcripts that were increased by MYOCD (FDR < 10^–5^) in the differential expression analysis. There were 12 transcripts in the overlay (Online resource 2F). *MBNL1*, *RBFOX2*, and *RBPMS* were again represented. We concluded that *MBNL1*, *RBFOX2*, and *RBPMS* are robustly regulated at the mRNA level by MYOCD, but so are several other RNA-binding proteins with a potential role in SMC-specific splicing.

### MYOCD drives alternative isoforms with genome-wide functional consequences

We next used the RNA-seq data to analyze transcript diversity and splicing. Exon-based analysis (DEXSeq) identified 5836 exonic regions showing differential usage. These regions covered 1637 features that contained 2677 genes (gene-level FDR = 0.01, Online resource 3). Identified genes were enriched for KEGG pathways that included “ribosome” and “focal adhesion”, and such GO terms as “structural constituent of muscle”, “extracellular matrix”, and “actin cytoskeleton”, (Online resource 4A). Event-based analysis (rMATS) isolated splicing events that included skipped exons (SE), alternative 5’ splice sites (A5SS), alternative 3’ splice sites (A3SS), mutually exclusive exons (MXE), and retained introns (RI, Online resource 3). There were 239 significant events (FDR < 0.05, |ΔPSI|> 10%) involving 190 genes. The distribution of these events across categories is depicted in Online resource 4B. Isoform-based analysis (*IsoformSwitchAnalyzeR*), finally, identified 515 genes (Online resource 3) with isoform switches and 151 (29%) of them had predicted functional consequences. There was an overall increase in exon skipping, intron retention, alternative 5´end donor site, alternative 3´end acceptor site, while alternative transcription starts and stops decreased. Analysis of functional consequences revealed significant over-representation of open reading frame (ORF) gain, protein domain gain, intrinsically disordered region (IDR) gain, intron retention (IR) gain, and “transcript is coding” (Online resource 4C).

Several genes overlapped between the three methods. The rMATS genes overlapped by 30% with the DEXSeq genes and by 6% with the IsoformSwitchAnalyzeR genes. Among the IsoformSwitchAnalyzeR genes, 36% were shared with the DEXSeq results. Overlaps at the exon level showed strong and significant correlation between the DEXSeq fold change and rMATS inclusion difference for intron retention and skipped exons (Online resource 4D and E). 12 genes were represented in all analyses and among them were *LRRC17*, *MID1*, *DDAH1*, *FLNA*, and *DMD*. 303 genes were represented in at least two of the analyses, and *MYLK*, *ACTN1*, *FN1*, *MYL9*, *VCL*, *PTPRF*, and *PPP1R12A* were among them. Thus, three different RNA-seq-based analysis methods support the view that MYOCD promotes transcript diversity and splicing.

### *MYLK* and *SLMAP* are likely examples of alternative transcription start sites favored by MYOCD

**Fig. 3:**
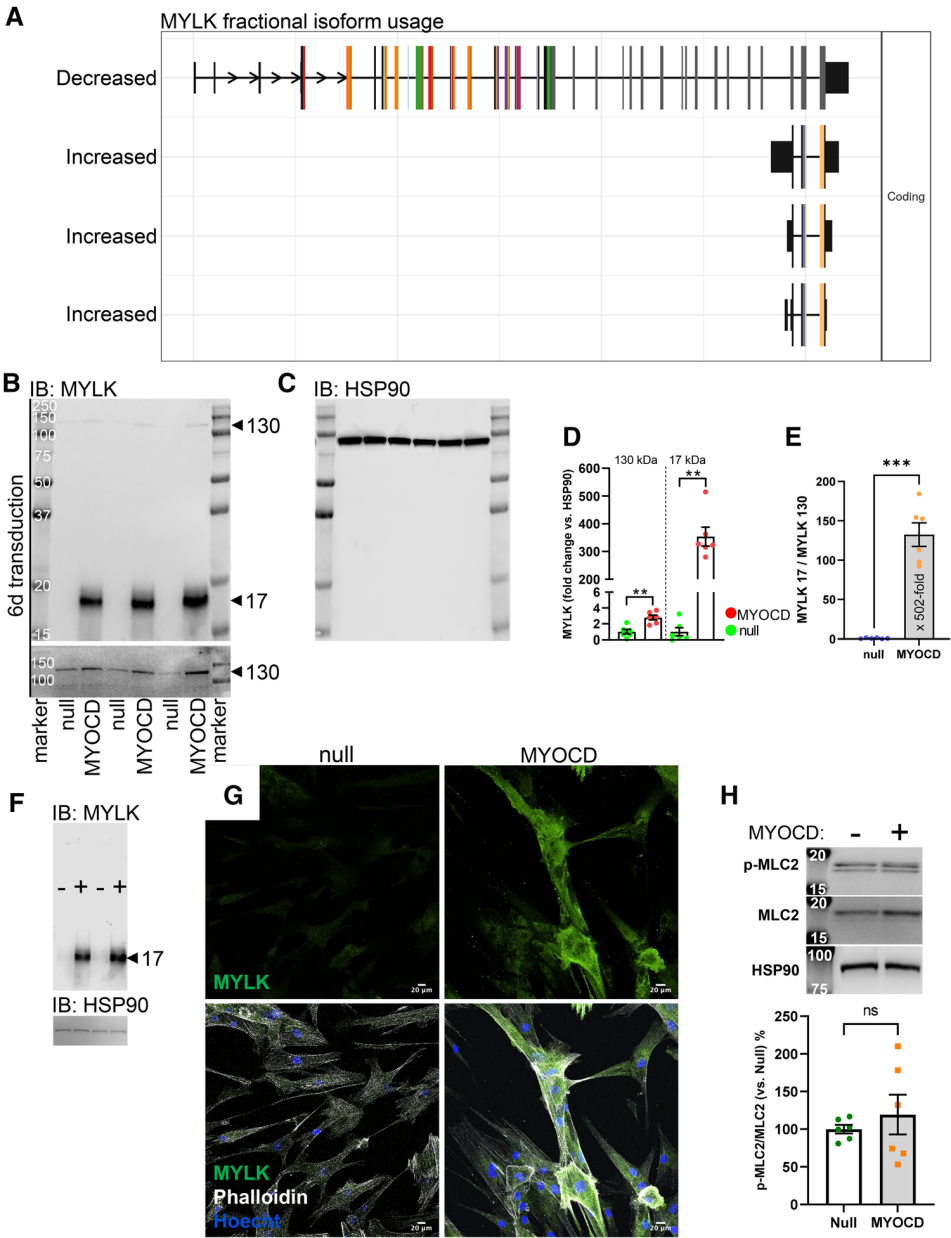
MYOCD promotes expression of telokin relative to myosin light chain kinase. *MYLK*, which encodes smooth muscle myosin light chain kinase (smMLCK) was captured by exon-based and isoform-based splicing analyses. The isoform-based analysis showed that three short isoforms derived from the 3´ end of the gene and that lack catalytic domains were favored by MYOCD at the expense of a longer transcript that included the catalytic domain (**A**). The colored exons represent different domains, and the kinase domain is encoded by the dark grey exons towards the 3´ end. Dark green, represent an intrinsically disordered-binding region, while red and orange colors represent IG-like domains. The short transcripts below the longest isoform represent three telokin-related peptides. Western blotting for MYLK (**B**) using MYOCD transduced coronary artery SMCs showed that a 130 kDa MYLK band increased modestly with MYOCD (B, D), while a 17 kDa band increased substantially (**B**, **D**). Faint bands at 19–20 and at 25 kDa were also increased by MYOCD (**B**). After blotting for MYLK, the membrane was stripped and re-probed for HSP90 (**C**) to ascertain equal protein loading. The MYLK17/MYLK130 ratio increased dramatically with MYOCD (**E**). Like coronary artery SMCs, MYOCD increased MYLK17 relative to MYLK130 in bladder SMCs (**F**), and imaging (**G**) indicted an overall increase of MYLK after MYOCD transduction. No striking difference in co-localization with actin filaments was apparent as shown using phalloidin to label filamentous actin. Myosin phosphorylation (P-MLC2) was not increased after MYOCD transduction as shown using western blotting (**H**)

We first sought to confirm examples of MYOCD-driven isoform diversity and focused on *MYLK*. *MYLK* encodes myosin light chain kinase (also called MLCK), the enzyme that mediates myosin phosphorylation and SMC contraction [57]. *MYLK* stood out in the DEXSeq results with a relative reduction of several 5´ exons and an increase of a handful of exons towards the 3´ end of the gene. *MYLK* was also among the top significant genes in the isoform-based results, showing a significant decrease of fractional usage for the longest isoform and corresponding increase in usage of three short isoforms that lack catalytic and actin-binding domains, while retaining the myosin binding domain at the C-terminus (Fig. 3A).

In mouse, *Mylk* generates three independent proteins due to alternative transcription start sites: 220 kDa myosin light chain kinase (MLCK), 130 kDa smooth muscle MLCK (smMLCK), and 17 kDa telokin (from a combination of the Greek telos, "end" and kinase) derived from the last three exons. These gene products are regulated by independent promoters [58–61]. The short *MYLK* transcripts that were increased by MYOCD in human coronary SMCs therefore likely represent telokin and telokin-related transcripts. Western blotting using a MYLK antibody detected a prominent 17 kDa band after overexpression of MYOCD that was absent in null samples (Fig. 3B). A band at 130 kDa also increased (see bottom of Fig. 3B for longer exposure), but no band was seen at 220 kDa. The MYLK blot was stripped and re-probed for a housekeeping protein (Fig. 3C) to allow for quantification. A modest but significant 2.8-fold increase was seen for the 130 kDa band, and the 17 kDa band increased several 100-fold (Fig. 3D), as did the ratio of the 17 and 130 kDa bands (Fig. 3E). Similarly, in human bladder SMCs, MYOCD increased telokin with only a moderate effect on 130 kDa MYLK such that the telokin/smMLCK ratio increased 7.7 ± 0.5-fold (*P* < 0.0001, *n* = 4, Fig. 3F). Inspection of ChIP-sec data (ENCODE) in the human genome browser revealed intronic SRF binding sites in human *MYLK*, two in the middle of the gene (in introns 7 and 12 of the longest transcript, hg19) and one just 5’ of the last exons (intron 29 of the longest transcript, hg19). These findings argue that telokin-related peptides represent the major proteins derived from the human *MYLK* locus following overexpression of MYOCD, and this likely depends on an internal promoter towards the end of the gene.

MYLK binds to actin via its N-terminal domain [62]. The C-terminal (telokin-) part of MYLK lacks a catalytic domain but is important for myosin binding. We therefore hypothesized that imaging for MYLK would show co-localization with actin in control conditions that is reduced after overexpression of MYOCD. Imaging using an antibody raised against a C-terminal MYLK peptide, and which therefore detects the 130 and 17 kDa isoforms (see above), showed an overall increase of fluorescence after MYOCD transduction. Association with actin was still apparent in some areas (Fig. 3G), and the antibody did not allow quantitative co-localization analysis, requiring cumbersome antigen retrieval. We also determined phosphorylation of the regulatory light chains of myosin (MLC2 encoded by *MYL9*). MYOCD did not increase the relative level of P-MLC2 (Fig. 3H), but interpretation is complicated by an increase of the MLC2 level with MYOCD. Functional consequences of preferential telokin regulation were thus difficult to ascertain in these experiments.

**Fig. 4:**
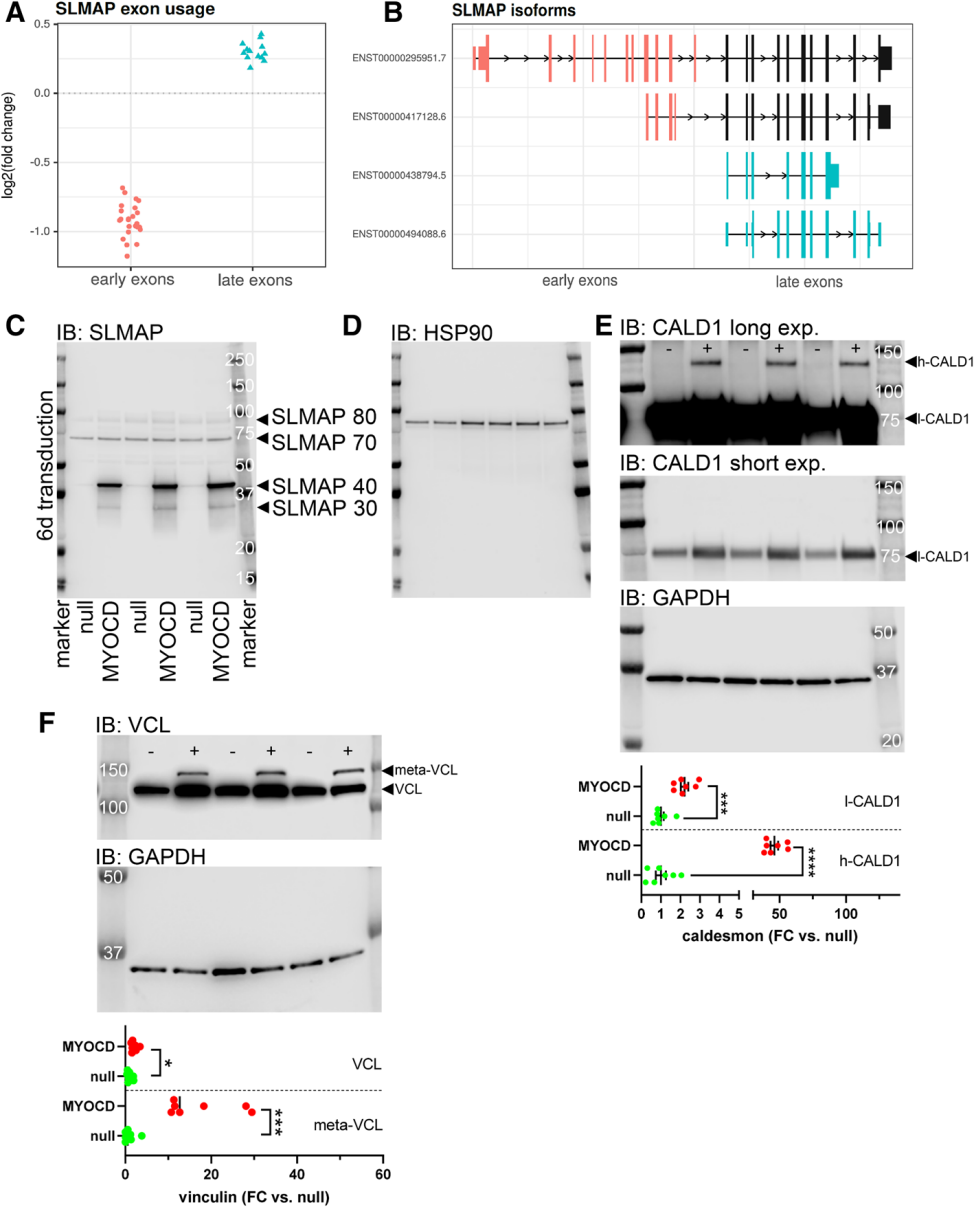
Additional examples of MYOCD-driven differential exon usage include *SLMAP*, *CALD1*, and *VCL*. *SLMAP* which encodes sarcolemma associated protein, was among the striking examples in the exon-based analysis. Reads mapping to 3´, late, exons were increased relative to early exons (**A**). Late exons are represented in the two annotated mRNA isoforms shown in light blue in (**B**). Western blotting showed SLMAP bands at 40 and 30 kDa that increased dramatically with MYOCD compared to null virus, while bands at 80 and 70 kDa remained inert (**C**). After blotting for SLMAP, the membrane was stripped and re-probed for HSP90 (**D**). Panel E shows western blotting for caldesmon (null: −; MYOCD: +). Heavy (h-CALD1) and light caldesmon (l-CALD1) variants were resolved, and intensity was adjusted to clearly show the heavy isoform in the top blot (long), and the lower band in the middle blot (short). Quantification, shown at the bottom, demonstrated a greater relative increase of h-CALD1 compared to l-CALD1. MYOCD similarly promoted expression of meta-vinculin (**F**), which is a SMC-enriched ≈ 150 kDa splice variant derived from the *VCL* gene, relative to vinculin (≈ 130 kDa)

Another transcript with a large difference in exon usage after MYOCD transduction was sarcolemma associated protein (*SLMAP*, gene level FDR = 0), which has an undefined role in blood pressure control and airway resistance [63, 64]. The DEXSeq analysis revealed decreased usage of early exons relative to late exons (Fig. 4A). *SLMAP* was not recovered in the isoform-based analysis, presumably due to considerable isoform complexity, so isoform assignment (Fig. 4B) remains hypothetical. Western blotting disclosed two bands at 40 and 30 kDa whose intensity changed 68 ± 4-fold and 22 ± 2-fold upon overexpression of MYOCD (Fig. 4C, D). Reasonably strong bands at 80 and at 70 kDa were not affected (1.1 ± 0.1-fold and 1.3 ± 0.1-fold changes). Encode ChIP-seq data indicates intronic SRF binding in *SLMAP* suggesting independent internal promoters driving late exons.

Taken together, these findings suggest that MYOCD regulates *MYLK* and *SLMAP* by activating internal promoters to drive expression of isoforms containing only the C-terminal regions present in full length isoforms.

### MYOCD promotes expression of SMC-specific isoforms of caldesmon and vinculin

We next focused on two SMC-specific splicing events [35]. The *CALD1* gene generates heavy (h-caldesmon: 135 kDa) and light (l-caldesmon: 75 kDa) caldesmon, and the heavy isoform is considered specific for SMCs. Western blotting showed that h-caldesmon increased 46 ± 3-fold while l-caldesmon increased only 2.2 ± 0.2-fold after MYOCD transduction (Fig. 4E). Consequently, the h- to l-caldesmon protein ratio increased 22 ± 0.9-fold (*P* < 0.001). Another example was *VCL*, which generates vinculin (130 kDa) along with the heavier SMC-enriched splice variant called meta-vinculin (150 kDa). Meta-vinculin was undetectable in coronary artery SMCs in control conditions, but a sharp band at the expected molecular weight of meta-vinculin was seen after overexpression of MYOCD (Fig. 4F). Therefore, the meta-vinculin to vinculin ratio increased 8.9 ± 0.6-fold (*P* < 0.001) with MYOCD. Taken together, these examples validate our RNA-seq analyses and argue that MYOCD promotes an exon usage and splicing code specific for SMCs.

### RBPMS-dependent splicing events driven by MYOCD

**Fig. 5:**
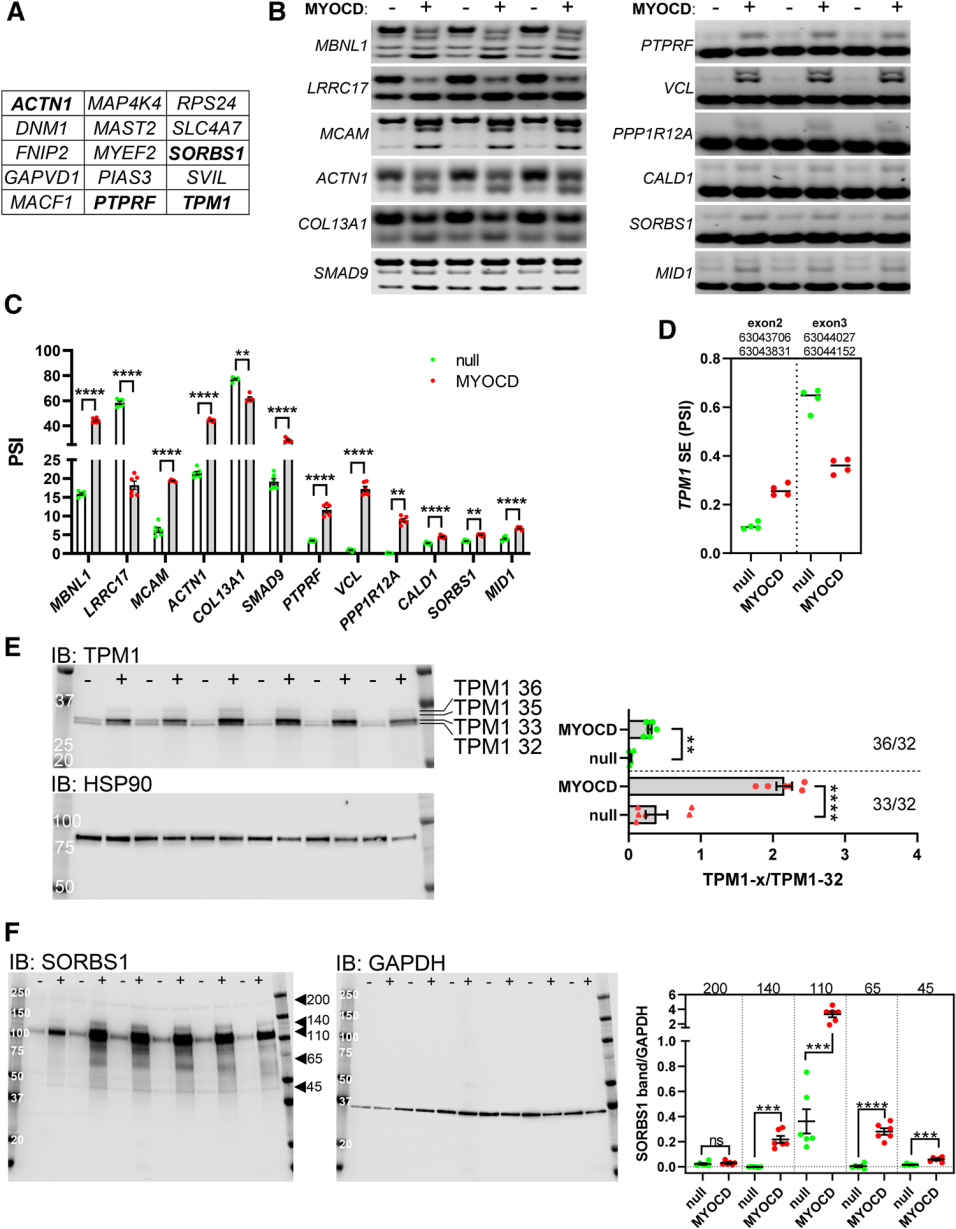
Event-based splicing analysis highlights potential RBPMS-dependent events downstream of MYOCD. The output from our rMATS analysis was overlapped with genes found to undergo Rbpms-dependent splicing in rat cells in a previous study [35]. **A** Genes from the overlap of these datasets. For confirmation of true splicing events, we used event-specific primers and PCR. Amplicons were separated on agarose gels (**B**, *n* = 5–6). Events are ordered by absolute change in percent spliced in (PSI). **C** Compiled data from panel B. TPM1, known to undergo SMC specific splicing, was represented in many categories in the rMATS analysis, showing increased usage of exon 2 and reduced usage of exon 3 (**D**). In panel E, TPM1 protein variants were examined by western blotting showing bands at 36 and 33 kDa that increased relative to other bands. Quantification is included in the bar graph on the right. Panel F shows western blotting for SORBS1. At least five SORBS1 protein species were detected and quantified (right)

Next, to get a better handle on splicing events regulated by MYOCD in an RBPMS-dependent manner, we focused on 92 events involving 65 genes that were common to Rbpms over-expression and knock down [35], and overlapped those with the genes from the current rMATS output. There were 15 genes represented in the overlap of these datasets, including *ACTN1*, *PTPRF*, *SORBS1*, and *TPM1* (Fig. 5A). *CALD1* and *VCL* depend on RBPMS for splicing in rat, but they are missing from the list (Fig. 5A) because they were not captured by rMATS (only by DEXSeq).

We thereafter selected splicing events for confirmation by PCR and agarose gel electrophoresis. Some of the results from these experiments are illustrated in Fig. 5B (*n* = 5–6). Among the chosen events, the largest shifts were seen for *MBNL1*, *LRRC17*, *MCAM*, *ACTN1*, and *COL13A1*. For example, MYOCD promoted skipping of 36 bp and 95 bp alternative exons in *MBNL1* (Fig. 5B). This is identical to the effect of Rbpms overexpression in rat SMCs [35]. Other switches were quantitatively smaller as exemplified by *SORBS1*, and *CALD1* (Fig. 5B, C). A functionally interesting example was the myosin phosphatase (MYPT1, encoded by the gene *PPP1R12A*). The exon inclusion detected is important for cyclic nucleotide dependent smooth muscle relaxation and blood pressure regulation [32, 65, 66]. MYOCD promoted inclusion (Fig. 5B, *PPP1R12A*), and there was essentially no inclusion in basal conditions. About one third of the event-specific primers resulted in more than two bands. In most cases, this is due to involvement of > 1 exons (e.g., *MBNL1*), but we did not explore the identity of additional bands throughout. We found no example among the confirmed events that favored the non-SMC variant. Compiled results from these confirmatory splicing assays are shown in Fig. 5C.

Figure 5D defines the architecture of the *TPM1* event involving exon 2 and exon 3. An increase of exon 2 usage and a reduction in usage of the mutually exclusive exon 3 was seen. Because the exon numbering is ambiguous, exact genomic coordinates are given for the respective exons/events (Fig. 5D, top). The reciprocal increase of exon 2 and reduction of exon 3 is consistent with the splicing pattern in mature SMCs [25]. The same is true for the *ACTN1* event, which involves switching from a mutually exclusive exon encoding a Ca^2+^-binding EF-hand to a variant that does not bind Ca^2+^, as observed in mature SMCs. In all, these analyses confirm that MYOCD favors SMC-specific alternative splicing of numerous mRNAs.

Some of the events were associated with differences in protein migration in western blot experiments. This is exemplified by TPM1 (Fig. 5E), which was represented in the MXE, A5SS, and SE categories of the rMATS analysis. Four bands between 36 and 32 kDa were resolved, and the first (36 kDa), second (35 kDa), and third (33 kDa) bands changed relative to the fourth (32 kDa) band on overexpression of MYOCD; quantitative analysis of the second band (35 kDa) was difficult, presumably because the stronger bands above and below contributed to the background, but analysis of the 36 and 33 kDa bands relative to the 32 kDa band is shown in the bar graph to the right. Another example was *SORBS1* (Fig. 5F) which was represented in the MXE category and that was robustly upregulated. Western blotting for SORBS1 showed a band at 200 kDa that remained static, while bands at 140, 110, 65, and 45 kDa increased. Bands at 140 and 65 kDa were absent in control conditions but were readily detectable after overexpression of MYOCD (Fig. 5F). We do not rule out that some of this complexity arises for reasons other than splicing, such as alternative internal promoters. Some splicing events were not associated isoform changes in western blotting, as exemplified by *ACTN1* (which however increased, Online resource 5A), *FLNA* (Online resource 5B), and *MBNL1* (see below).

### A putative RBPMS/RBFOX2 complex downstream of MYOCD

Our experiments so far allowed us to refine the hypothesis formulated at the outset of this work. That is, MYOCD promotes expression of RBPMS and RBFOX2, and this in turn favors SMC-specific splicing. Based on this refined hypothesis, we made three predictions. The first prediction was that binding motifs for RBPMS and RBFOX2 should be enriched around exons that are activated or repressed by MYOCD. The second prediction was that silencing of RBPMS or RBFOX2 should antagonize the effect of MYOCD on splicing. Finally, because MYOCD, but not MRTF-A, regulated RBPMS (c.f. Figure 2C), we also predicted that the effect of MYOCD on splicing should not be shared by MRTF-A.

We first examined occurrence of binding motifs for splicing factors around the alternatively spliced exons identified by rMATS. We focused on motifs for RBPMS defined as CACn(1–12)CAC (Online resource 6, top), RBFOX defined as GCAC/TG (Online resource 6, middle), and MBNL defined as C/TGCC/T (Online resource 6, bottom). Each of these proteins has previously been demonstrated to have position-dependent activity, activating exon inclusion when binding downstream of target exons, but inhibiting when bound upstream or within the exon. RBFOX motifs were similarly enriched downstream of exons that are activated by MYOCD and upstream and within exons that are repressed, suggesting that MYOCD expression activates a program of RBFOX regulated events. RBPMS and MBNL motifs were associated only with exons that showed increased skipping upon MYOCD expression, and the motifs were enriched at various locations in both flanking introns. Therefore, motif enrichment favored RBFOX2 as an important driver of AS following overexpression of MYOCD, with RBPMS and MBNL1 potentially also playing important roles.

**Fig. 6:**
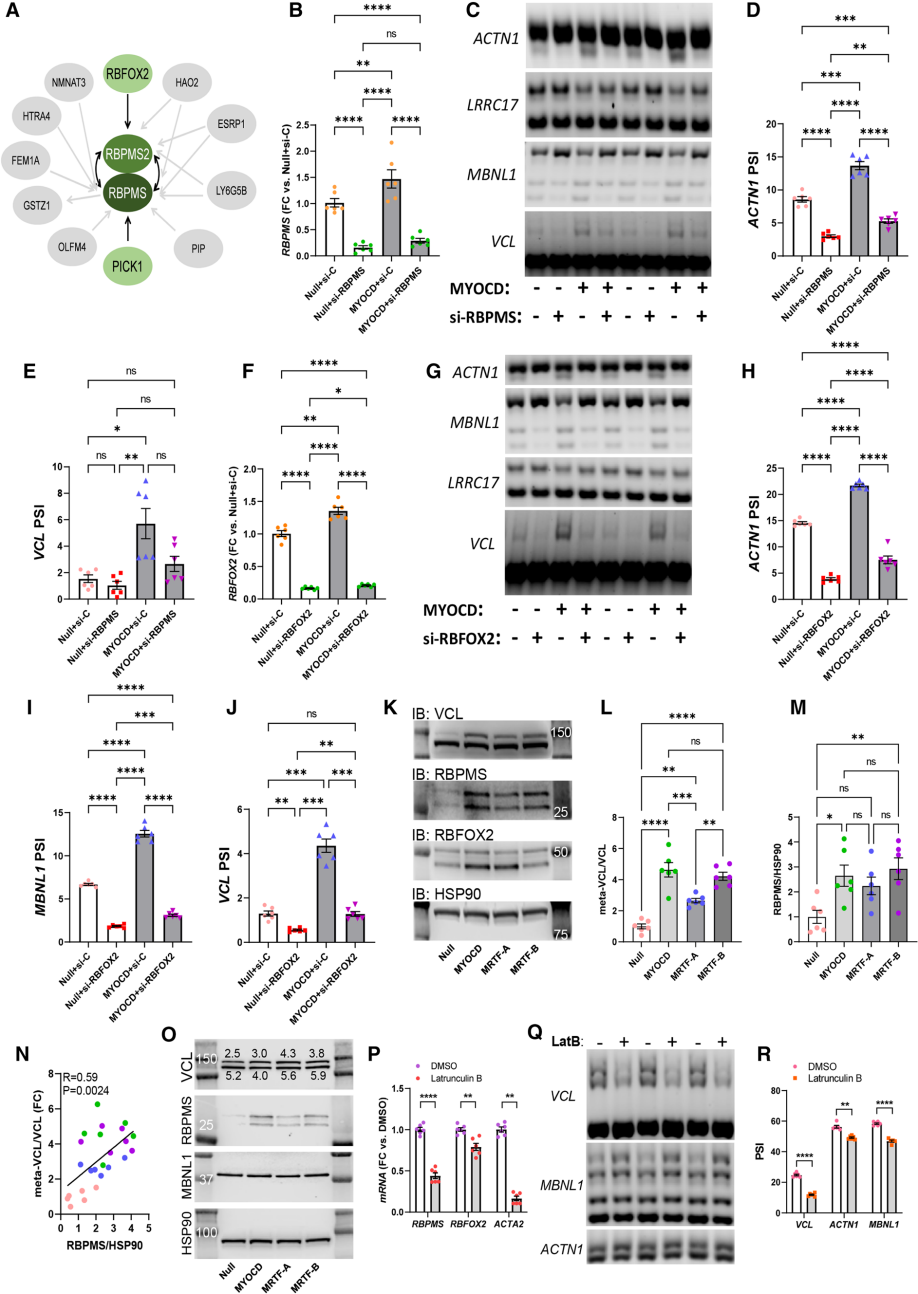
RBPMS RBFOX2 act downstream of MYOCD. A previous study identified a putative splicing complex consisting of RBPMS, RBPMS2, and RBFOX2 (**A)**. We noted that an additional partner in this complex (PICK1) was regulated by MYOCD at the mRNA level, suggesting that MYOCD exerts its splicing effects via 3–4 core partners (green) in a splicing factor complex. Silencing of RBPMS (**B**), antagonized MYOCD-driven changes in splicing of *ACTN1*, *MBNL1*, and *VCL* as shown using variant-specific primers and agarose gel electrophoresis (**C**–**E**, *n* = 6 in this and the following panels). Similarly, silencing of RBFOX2 (**F**), antagonized the effect of MYOCD on splicing of these transcripts (**G**–**J**). We also examined if MYOCD, but not MRTF-A, can drive differential splicing of vinculin (VCL) using western blotting (**K**). All MRTFs increased the meta-VCL/VCL ratio (**L**). RBPMS was preferentially regulated by MYOCD and MRTF-B (**M**), but RBPMS and the meta-VCL to VCL ratio correlated across the entire dataset (**N**). All MRTFs similarly promoted VCL splicing in bladder SMCs (**O**, numbers from the densitometric quantification are shown by the bands). Because MRTF-A and MRTF-B are inhibited by depolymerization of actin, bladder SMCs were treated with Latrunculin B, which depolymerizes actin. This reduced the mRNA levels of RBPMS and RBFOX2 (**P**) and inhibited SMC-specific splicing (**Q**, **R**)

Affinity purification/mass spectrometry has identified RBFOX2 as an interaction partner of RBPMS and RBPMS2 [67] (Fig. 6A). We realized that an additional partner in this complex, *PICK1*, is regulated at the mRNA level by MYOCD. This suggested that MYOCD regulates three to four partners in an RBPMS/RBFOX2 splicing complex. We therefore examined if knocking down either RBPMS or RBFOX2 would antagonize effects of MYOCD on splicing. Figure 6B shows successful silencing of *RBPMS* in the absence (white bars), and presence (grey bars) of MYOCD. *RBPMS* silencing reversed the effect of MYOCD on splicing of *ACTN1*, *MBNL1*, and *VCL* (Fig. 6C–E, and Online resource 5C). Silencing of *RBFOX2* was also successful (Fig. 6F), and this again impaired MYOCD’s ability to drive splicing (Fig. 6G-J). Some of the splicing events promoted by MYOCD therefore depend on both RBPMS and RBFOX2.

Our last prediction was that MYOCD, but not MRTF-A, should drive an RBPMS-dependent splicing event. To examine this prediction MYOCD was overexpressed in parallel with MRTF-A and MRTF-B (8 days) and we examined the appearance of meta-vinculin by western blotting (Fig. 6K, L). Contrary to our prediction, both MYOCD and MRTF-A increased the meta-vinculin to vinculin ratio (Fig. 6L). We therefore examined RBPMS and RBFOX2 in the same lysates. RBPMS was increased by MYOCD and MRTF-B (Fig. 6M), but not significantly by MRTF-A (P = 0.12, ANOVA-Tukey). However, the level of RBPMS in MRTF-A transduced cells was not different from that in cells transduced with MYOCD or MRTF-B, and the RBPMS level correlated with vinculin splicing across the entire dataset (Fig. 6N, Spearman, pink dots = null, blue = MRTF-A, green = MYOCD, purple = MRTF-B). This was not seen for RBFOX2 (*R* = 0.21, *P* = 0.33, Spearman). Taken together, this suggested that RBPMS induction with MRTF-A is just marginally smaller than with MYOCD, but nonetheless sufficient to drive splicing. MRTF-A also promoted vinculin splicing in bladder SMCs (Fig. 6O, *P* ≤ 0.036 for all co-activators, ANOVA-Dunnett), and this appeared to correlate with its effect on RBPMS. MBNL1 was unaffected (Fig. 6O).

Contrasting with MYOCD, which is constitutively nuclear, the physiological control mechanism for MRTF-A and MRTF-B involves changes in actin polymerization. Having established that MRTF-A and MRTF-B affect splicing of vinculin like MYOCD, we next asked if depolymerization of actin would reduce SMC-specific splicing. For this we used Latrunculin B (LatB) which depolymerizes actin. LatB treatment can be toxic to cells, but we used a protocol where cells recover for 4 h without LatB every 24 h [55]. We focused on bladder SMCs in these experiments because they contain more meta-vinculin than coronary SMCs to start with (compare Fig. 6O null with 6 K null), making it more likely that we would see a difference with LatB. Depolymerization of actin reduced *RBPMS* and *RBFOX2*, along with the positive control *ACTA2* (Fig. 6P). This associated with reduced SMC-specific splicing of *VCL, MBNL1*, and *ACTN1* as shown by PCR and agarose gel electrophoresis (Fig. 6Q, R). Similar, but less pronounced results were obtained in coronary artery SMCs (Online resource 5F, G). Interestingly, and in contrast to coronary artery SMCs, SMC-specific splicing of MBNL1 dominated in bladder SMCs (compare Fig. 6Q, G). This is consistent with the more pronounced SMC-specific splicing of *VCL* (Fig. 6O vs. K) and *ACTN1* (Fig. 6Q vs. C) in bladder versus coronary artery SMCs in culture.

### Srf knockout affects SMC-specific splicing in vivo

**Fig. 7:**
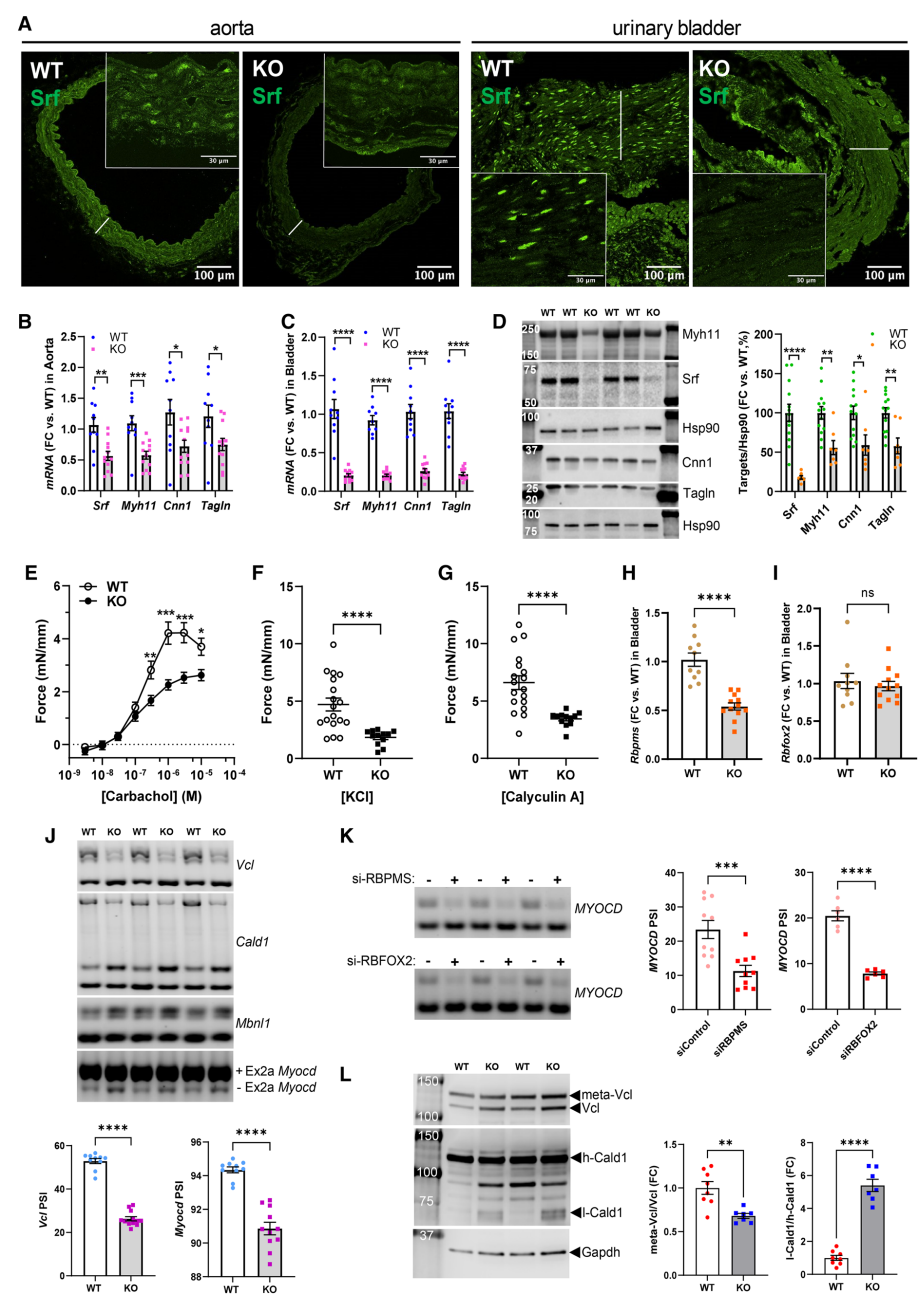
Inducible and SMC-specific knockout of Srf in vivo reduces SMC splicing of *Vcl*, *Cald1*, and *Myocd*. To address the in vivo relevance of Myocd-Srf-driven splicing, we generated mice allowing for inducible deletion of Srf in SMCs (Myh11-CreER^T2^ x Srf^fl/fl^ mice injected with tamoxifen, referred to as knockout, KO). Compared to wild type mice (WT), staining for Srf (green), which is a predominately nuclear protein, was reduced in SMCs in the aorta and urinary bladder (**A**). White lines in the micrographs highlight the SMC layers of the aorta and bladder, respectively. This was confirmed by RT-qPCR, showing reduction of *Srf* along with three contractile markers in both aorta (**B**), and bladder (**C**). We focused on the bladder in view of better Srf depletion and could confirm sizeable reduction of Srf and SMC markers by western blotting (**D**). Contraction in response to cumulative addition of the muscarinic agonist carbachol (**E**), depolarization with KCl (60 mM, **F**), and stimulation with the phosphatase inhibitor Calyculin A (1 µM, **G**), was reduced, confirming loss of contractility in KO vs. WT bladder. In addition to these expected phenotypes, we also observed a reduction of *Rbpms* (**H**), but not of *Rbfox2* (**I**) by RT-qPCR. Splicing of *Vcl*, *Cald1*, and *Mbnl1* was altered as shown using PCR and agarose gel electrophoresis (**J**). Myocd splicing (exon 2a) was also examined. Myocd splicing changed from the SMC variant containing exon 2a (+ Ex2a), towards a variant that lacks exon 2a (− Ex2a, panel J, bottom). Silencing of either RBPMS or RBFOX2 similarly favored the heart variant (− Ex2a) of *MYOCD* in cultured human coronary SMCs (**K**). Altered splicing of Vcl, and Cald1 in KO bladder was confirmed by western blotting (**L**)

To examine if MRTF-SRF signaling influences SMC splicing in vivo we used two strategies. First, we used mice with inducible and SMC-specific knockout of Srf. Inducible knockout of Srf in SMCs leads to death from gastrointestinal complications 28 days after induction with tamoxifen. Here, we harvested the aorta and urinary bladder from knockout (KO) and wild type (WT) mice at 21–25 days. Nuclear Srf staining was reduced in the SMC layers of both the aorta and bladder from knockout mice, but the difference appeared more pronounced in bladder (Fig. 7A, right, insets show high magnification views). Similarly, reduction of Srf and contractile markers (*Myh11*, *Cnn1*, and *Tagln*) at the mRNA level were more pronounced in bladder (*Srf* reduced by 81%, Fig. 7C) than in aorta (*Srf* reduced by 47%, Fig. 7B). We thus focused on the bladder. In the KO bladder, Srf and the contractile markers were reduced at the protein level (Fig. 7D), and contraction in response to the muscarinic agonist carbachol (Fig. 7E), depolarization (Fig. 7F), and myosin phosphatase inhibition (Fig. 7G) was reduced compared to WT mice. Knockout of Srf in vivo therefore leads reduced contractile differentiation and to loss of contractility. In keeping with our hypothesis, reduction of *Rbpms* was moreover observed in Srf KO mice (Fig. 7H), while *Rbfox2* was unaffected (Fig. 7I). Agarose gel electrophoresis demonstrated altered splicing of *Vcl*, *Cald1*, and *Mbnl1* as anticipated (Fig. 7J). Because *Myocd* splicing is Rbpms-dependent in rat SMCs [35], and given that it was pointless to examine MYOCD splicing in the previous experiments where MYOCD was overexpressed, we also examined splicing of *Myocd* in Srf KO bladder. Exclusion of *Myocd* exon 2a, which produces a longer Myocd protein that predominates in heart [33], increased in KO compared to WT bladder (Fig. 7J, bottom gel). Moreover, in human coronary SMCs, silencing of either RBPMS or RBFOX2, increased the heart variant of MYOCD at the expense of the SMC variant (Fig. 7K). In contrast to bladder tissue, where the SMC isoform of *Myocd* dominated (≈95% + exon 2a, Fig. 7J), the SMC isoform was the least abundant isoform in cultured SMCs (≈25% + exon2a, Fig. 7K), consistent with reduction of contractile differentiation in culture. We also examined Vcl and Cald1 splicing at the protein level in Srf knockout bladder, and SMC-specific splicing was reduced in both cases (Fig. 7L), even if the Cald1 splicing pattern was more complex in mouse bladder than in human SMCs (compare Fig. 7L with Fig. 4E).

Second, rather than using all reads mapping to a gene (as in Fig. 1), we used different features (transcript isoforms, exon reads, and junction reads) for correlations with the overall level of *MYOCD*. We observed correlation patterns consistent with the differential exon usage documented for MYOCD transduced SMCs in case of *TPM1*, *SLMAP*, *VCL*, *ACTN1*, *LRRC17* (Online resource 7). For instance, expression of transcripts and junctions with exon 2 in *TPM1* correlated more strongly with *MYOCD* than did expression of transcripts and junctions including exon 3. Similarly, the pattern of down-regulation of early exons relative to late exons in *SLMAP* upon *MYOCD* transduction, was reflected in the pattern of correlation of early and late exons with *MYOCD*. Correlation patterns for *MYLK*, *MBNL1*, and *PPP1R12A* were less conclusive, presumably due to more complex splicing structures and a high number of transcripts. In the same manner we examined if features including exon 2a in *MYOCD* correlated better with the overall *RBPMS* level than did features skipping exon 2a. Junction level correlations showed this pattern, but exon level correlations did not (Online resource 8).

**Fig. 8:**
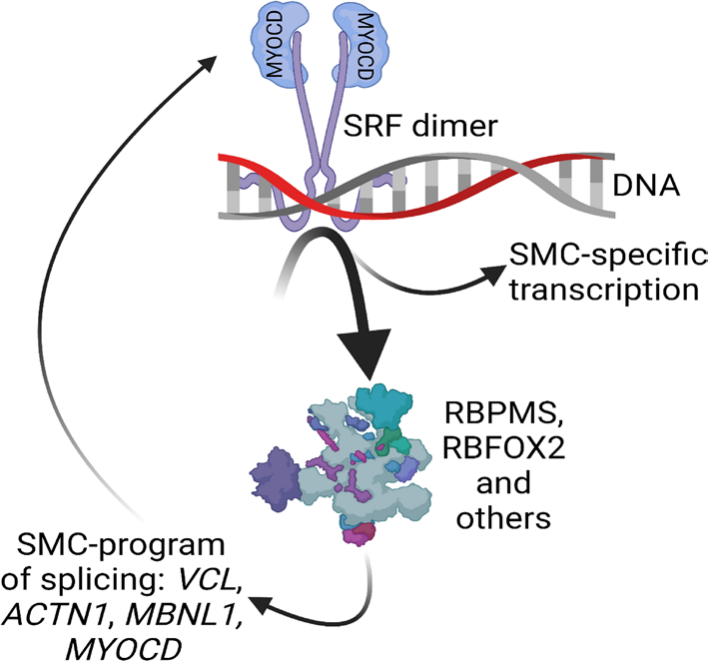
A model for MYOCD-driven splicing in SMCs. Cartoon depicting the model suggested by this work, where MYOCD targets splicing regulators through SRF, including RBPMS, to promote SMC-specific splicing of *VCL*, *ACTN1*, and others. This would appear to include *MYOCD* itself. Given that the SMC variant of MYOCD (+ Ex2a) may be a more effective co-activator of SMC genes, this may result in a positive feedback loop, allowing for slow maturation of SMCs. Aspects of this model require further experimental verification. The cartoon was generated using BioRender

Taken together, our work favors a model where activity of MYOCD and SRF drives SMC-specific splicing of several targets, in part through RBPMS, including *VCL* and *MYOCD* itself, as depicted in Fig. 8.

## Discussion

The present work demonstrates that MYOCD regulates splicing factors and influences splicing in human SMCs. Moreover, MYOCD impacts proteome diversity via alternative promoters and transcription start sites as well as alternative 3’ ends. Unearthing of RBPMS in the MYOCD co-expression module, and the novel finding that RBPMS is regulated by MYOCD at the mRNA and protein levels is of particular interest given the recent identification of Rbpms as a regulator of splicing in rat SMCs [35]. That study also provided evidence that Rbpms is reduced upon modulation of SMCs towards the synthetic phenotype, in good agreement with how *MYOCD* behaves in cell culture [3, 68, 69]. Rbpms is involved in numerous splicing events, involving e.g., *Vcl*, *Mbnl1*, and *Actn1* [35]. Our demonstration that SMC-specific splicing changes involving these transcripts occur after MYOCD transduction is consistent with the view that RBPMS is downstream of MYOCD. Moreover, the events identified are identical to the events driven by Rbpms in rat SMCs [35]. We also demonstrate that silencing of RBPMS abrogates the effect of MYOCD on several of these alternative splicing events. Taken together, these are compelling arguments that MYOCD exerts some, but likely not all, of its effects on splicing through RBPMS.

Additional splicing factors in the MYOCD co-expression module are *MBNL1*, *RBPMS2*, and *RBFOX2*. Because RBPMS and RBFOX2 bind to each other [67], and given that silencing of either of them impairs MYOCD-driven splicing, it makes sense to suggest that the effects of MYOCD on splicing are funneled in part by an RBPMS/RBFOX2 complex. This would also explain why our motif enrichment analysis favored RBFOX2 over RBPMS, despite the stronger correlation between RBPMS expression and splicing. Moreover, exons that are regulated in response to RBPMS knockdown or overexpression in rat SMCs [35], are associated not only with binding motifs for RBPMS but also RBFOX (AJ, unpublished observations). We believe that changes in other splicing factors, such as the reduction of QKI, are also relevant for the transcriptome-wide changes documented here, but protein level data in those cases was not generated. We did examine the MBNL1 protein, but neither its overall level nor its isoform distribution changed in MYOCD transduced cells. This was surprising for at least two reasons. One is that a recent study reported that MYOCD regulates the expression of MBNL1 at the mRNA and protein level via a CArG box in the proximal promoter [70]. Another is that Rbpms controls the Mbnl1 protein isoform distribution in rat SMCs [35]. Given that MYOCD promoted skipping of 36 bp and 95 bp exons in *MBNL1*, identical to the effect of Rbpms in rat SMCs, but left downstream secondary events unaffected (such as *LSM14B* splicing [35], Online resource 3), it seems likely that at least the effect on MBNL1 protein isoform distribution would have become evident with longer times of transduction. We do not rule out that the discrepancy between MBNL1 mRNA and protein arises for other reasons than time of transduction, such as increase of the antisense transcript *MBNL1-AS1*, which was the most MYOCD-responsive long non-coding RNA in our sequencing experiment.

Two observations argue that the effects of MYOCD and MRTF-B on RBPMS are direct and mediated by SRF. First, we find that SRF knockdown reduces the basal level of *RBPMS* and that it antagonizes the effect of MRTF-B. Second, chromatin immunoprecipitation data in the genome browser (ENCODE) indicates SRF binding close (≈ 2 kb) to the transcription start site of the longest transcript, and within two introns. Single nucleotide polymorphisms are scattered across the RBPMS locus and in close vicinity to the SRF binding sites, but these associate primarily with hematological phenotypes, including e.g., platelet count. Knockout models for Rbpms and Rbpms2 have been created by the International Mouse Phenotyping Consortium (https://www.mousephenotype.org/), and numerous phenotypes have been recorded. For instance, knockout of Rbpms leads to pre-weaning lethality with 100% penetrance. A recent study using an independent Rbpms knockout mouse reported complete lethality by postnatal day 4, associated with patent ductus arteriosus and noncompaction cardiomyopathy [71]. Tissue-specific and inducible strategies to address the in vivo significance of RBPMS in SMC function are thus warranted.

One of the most notable events of differential exon usage detected here involves *MYLK*, which encodes 130 kDa myosin light chain kinase (MLCK) and 17 kDa telokin. Knockout of the intronic telokin promoter in mouse leads to loss of telokin expression and increased smooth muscle contraction [59]. This is opposite to the effect seen after knockout of smooth muscle MLCK, which leads to impaired contraction [72]. Two of our analyses showed that 3’ exons in *MYLK* are preferentially expressed following transduction of MYOCD, and this associates with appearance of a 17 kDa telokin band. Moreover, SRF binds internal sequences in the human *MYLK* gene. In all, our results therefore support differential regulation of human telokin and MLCK by two internal MYOCD-driven promoters with rather different strengths. Mouse telokin is activated tenfold [60] and MLCK 25-fold [61] by Myocd, but we find here that human telokin is activated 350-fold and MLCK only threefold (human coronary SMCs). Thus, the major MYOCD-driven products of the human *MYLK* gene are telokin and two telokin-like peptides.

MYOCD drives several other events of AS and differential exon usage that are likely to be functionally relevant. In the case of *ACTN1*, the splicing event detected abrogates Ca^2+^-binding. The proposed relevance is that dynamic and Ca^2+^ dependent cross-linking of actin filaments, while important in motile cells, is not needed in contractile SMCs in tissue [73]. Specific targeting strategies have been used for knockout of h-caldesmon, and this influences rate of relaxation [74]. Meta-vinculin, finally, was shown to affect force transduction at cell adhesion sites [75]. The event involving MYPT1 (*PPP1R12A*), which encodes the myosin phosphatase targeting subunit, affects nitric oxide-dependent arterial dilatation and blood pressure [31, 32]. An interesting observation in this case was that exon 24 inclusion was absent under basal conditions and seen only after overexpression of MYOCD. One would thus predict that disruption of MRTF-SRF signaling in vivo should impair nitric oxide-dependent dilatation. Exactly this has been reported [6], and the defect seems to be situated downstream of cGMP generation, consistent with the idea that myosin phosphatase activation [76] falters.

Our experiments with different myocardin-related transcription factors did not support critical differences between them in relation to splicing. In fact, their effects on RBPMS expression correlated with their effect on vinculin splicing. Accordingly, we found that depolymerization of actin also influenced splicing. This finding is of interest because the physiological activation mechanism of MRTF-A and MRTF-B involves actin dynamics, such as caused by mechanical forces and geometrical constraints. One may thus speculate that a differential effect of MYOCD and MRTF-A on splicing is not a major reason why MRTF-A cannot compensate for MYOCD in smooth muscle homeostasis [7].

We demonstrate that SMC-specific and inducible knockout of Srf leads to reduction of *Rbpms* in the bladder, and this associates with altered splicing of *Vcl*, *Cald1*, and *Myocd* itself. In addition to supporting the in vivo relevance of Myocd-Srf-driven splicing, this may allow for a positive feedback mechanism. This is because the SMC variant of myocardin in some cases is a more effective co-activator of target genes [77]. Sustained myocardin expression may thus tentatively promote its own splicing (Fig. 8), eventually yielding a more effective co-activator of SRF.

Constraints imposed by bioinformatic techniques, use of short sequence reads, and sequencing depth can limit the number of splicing events detected. Simulations suggest that a sequencing depth of 50 million reads per sample results in the detection accuracy of 0.29–0.91 depending on the variation between samples [48]. We reached, on average, a sequencing depth of 52 million reads per sample and the variation between samples was small, suggesting that we targeted the higher end of this range. Even if some splicing events were missed, we still detected a considerable number of interesting genes with alternative splicing, and our results were validated using other methods. Studies of the functional impact of specific splicing changes driven by MYOCD is difficult at the tissue level in humans and were beyond the scope of the present work, but the current survey arguably makes the field better equipped to select such events for testing in model systems.

To conclude, the present work shows that MYOCD, which is critical for SMC gene expression and identity, implements a unique exon usage code beyond its effect on SRF-dependent transcription. This impacts isoform diversity at the mRNA and protein levels. MYOCD works through the splicing factors RBPMS and RBFOX2 to program exon usage, and this has remained an overlooked but critical aspect of SMC differentiation and function. Among the MYOCD-driven alternative transcripts, there are several which are important for SMC function. One of the most striking examples involves independent regulation of telokin-like peptides from the *MYLK* locus. These peptides lack catalytic domains and can therefore not activate myosin. The effect of MYOCD on splicing is at least partly shared among the MRTF family members. Accordingly, SMC splicing also responds to changes in actin dynamics.

### Supplementary Information

Below is the link to the electronic supplementary material.

Supplementary file1 (TIF 3580 KB)

Supplementary file2 (TIF 66266 KB)

Supplementary file3 (XLSX 2324 KB)

Supplementary file4 (TIF 67502 KB)

Supplementary file5 (TIF 9343 KB)

Supplementary file6 (TIF 61868 KB)

Supplementary file7 (XLSX 36 KB)

Supplementary file8 (XLSX 11 KB)

## Data Availability

Links to RNA-seq data, analyses, and code are given in materials and methods or as Online resource files. For example, RNA-Seq data is available at the Sequence Read Archive with the BioProject PRJNA731342. Data from other experiments are available from the lead or corresponding author on reasonable request.

## References

[1] Yap C, Mieremet A, de Vries CJM, Micha D, de Waard V (2021). Six Shades of Vascular Smooth Muscle Cells Illuminated by KLF4 (Kruppel-Like Factor 4). Arterioscler Thromb Vasc Biol.

[2] Owens GK, Kumar MS, Wamhoff BR (2004). Molecular regulation of vascular smooth muscle cell differentiation in development and disease. Physiol Rev.

[3] Chen J, Kitchen CM, Streb JW, Miano JM (2002). Myocardin: a component of a molecular switch for smooth muscle differentiation. J Mol Cell Cardiol.

[4] Miano JM (2015). Myocardin in biology and disease. J Biomed Res.

[5] Miano JM (2003). Serum response factor: toggling between disparate programs of gene expression. J Mol Cell Cardiol.

[6] Galmiche G, Labat C, Mericskay M, Aissa KA, Blanc J, Retailleau K, Bourhim M, Coletti D, Loufrani L, Gao-Li J, Feil R, Challande P, Henrion D, Decaux JF, Regnault V, Lacolley P, Li Z (2013). Inactivation of serum response factor contributes to decrease vascular muscular tone and arterial stiffness in mice. Circ Res.

[7] Huang J, Wang T, Wright AC, Yang J, Zhou S, Li L, Yang J, Small A, Parmacek MS (2015). Myocardin is required for maintenance of vascular and visceral smooth muscle homeostasis during postnatal development. Proc Natl Acad Sci USA.

[8] Angstenberger M, Wegener JW, Pichler BJ, Judenhofer MS, Feil S, Alberti S, Feil R, Nordheim A (2007). Severe intestinal obstruction on induced smooth muscle-specific ablation of the transcription factor SRF in adult mice. Gastroenterology.

[9] Johnson JM, Castle J, Garrett-Engele P, Kan Z, Loerch PM, Armour CD, Santos R, Schadt EE, Stoughton R, Shoemaker DD (2003). Genome-wide survey of human alternative pre-mRNA splicing with exon junction microarrays. Science.

[10] Wang ET, Sandberg R, Luo S, Khrebtukova I, Zhang L, Mayr C, Kingsmore SF, Schroth GP, Burge CB (2008). Alternative isoform regulation in human tissue transcriptomes. Nature.

[11] Nilsen TW, Graveley BR (2010). Expansion of the eukaryotic proteome by alternative splicing. Nature.

[12] E.P. Consortium (2012). An integrated encyclopedia of DNA elements in the human genome. Nature.

[13] Merkin J, Russell C, Chen P, Burge CB (2012). Evolutionary dynamics of gene and isoform regulation in Mammalian tissues. Science.

[14] Barbosa-Morais NL, Irimia M, Pan Q, Xiong HY, Gueroussov S, Lee LJ, Slobodeniuc V, Kutter C, Watt S, Colak R, Kim T, Misquitta-Ali CM, Wilson MD, Kim PM, Odom DT, Frey BJ, Blencowe BJ (2012). The evolutionary landscape of alternative splicing in vertebrate species. Science.

[15] Green ID, Liu R, Wong JJL (2021). The Expanding Role of Alternative Splicing in Vascular Smooth Muscle Cell Plasticity. Int J Mol Sci.

[16] Fu XD, Ares M (2014). Context-dependent control of alternative splicing by RNA-binding proteins. Nat Rev Genet.

[17] Pal S, Gupta R, Kim H, Wickramasinghe P, Baubet V, Showe LC, Dahmane N, Davuluri RV (2011). Alternative transcription exceeds alternative splicing in generating the transcriptome diversity of cerebellar development. Genome Res.

[18] Scotti MM, Swanson MS (2016). RNA mis-splicing in disease. Nat Rev Genet.

[19] Hasimbegovic E, Schweiger V, Kastner N, Spannbauer A, Traxler D, Lukovic D, Gyongyosi M, Mester-Tonczar J (2021). Alternative splicing in cardiovascular disease—a survey of recent findings. Genes (Basel).

[20] Tress ML, Abascal F, Valencia A (2017). Alternative splicing may not be the key to proteome complexity. Trends Biochem Sci.

[21] Sobue K, Hayashi K, Nishida W (1999). Expressional regulation of smooth muscle cell-specific genes in association with phenotypic modulation. Mol Cell Biochem.

[22] Llorian M, Gooding C, Bellora N, Hallegger M, Buckroyd A, Wang X, Rajgor D, Kayikci M, Feltham J, Ule J, Eyras E, Smith CW (2016). The alternative splicing program of differentiated smooth muscle cells involves concerted non-productive splicing of post-transcriptional regulators. Nucleic Acids Res.

[23] Nagai R, Kuro-o M, Babij P, Periasamy M (1989). Identification of two types of smooth muscle myosin heavy chain isoforms by cDNA cloning and immunoblot analysis. J Biol Chem.

[24] Babu GJ, Loukianov E, Loukianova T, Pyne GJ, Huke S, Osol G, Low RB, Paul RJ, Periasamy M (2001). Loss of SM-B myosin affects muscle shortening velocity and maximal force development. Nat Cell Biol.

[25] Gooding C, Roberts GC, Moreau G, Nadal-Ginard B, Smith CW (1994). Smooth muscle-specific switching of alpha-tropomyosin mutually exclusive exon selection by specific inhibition of the strong default exon. EMBO J.

[26] Gooding C, Edge C, Lorenz M, Coelho MB, Winters M, Kaminski CF, Cherny D, Eperon IC, Smith CW (2013). MBNL1 and PTB cooperate to repress splicing of Tpm1 exon 3. Nucleic Acids Res.

[27] Hayashi K, Fujio Y, Kato I, Sobue K (1991). Structural and functional relationships between h- and l-caldesmons. J Biol Chem.

[28] Tang ZZ, Liang MC, Lu S, Yu D, Yu CY, Yue DT, Soong TW (2004). Transcript scanning reveals novel and extensive splice variations in human l-type voltage-gated calcium channel, Cav1.2 alpha1 subunit. J Biol Chem.

[29] Hofmann F, Flockerzi V, Kahl S, Wegener JW (2014). L-type CaV1.2 calcium channels: from in vitro findings to in vivo function. Physiol Rev.

[30] Welling A, Ludwig A, Zimmer S, Klugbauer N, Flockerzi V, Hofmann F (1997). Alternatively spliced IS6 segments of the alpha 1C gene determine the tissue-specific dihydropyridine sensitivity of cardiac and vascular smooth muscle L-type Ca2+ channels. Circ Res.

[31] Khatri JJ, Joyce KM, Brozovich FV, Fisher SA (2001). Role of myosin phosphatase isoforms in cGMP-mediated smooth muscle relaxation. J Biol Chem.

[32] Reho JJ, Kenchegowda D, Asico LD, Fisher SA (2016). A splice variant of the myosin phosphatase regulatory subunit tunes arterial reactivity and suppresses response to salt loading. Am J Physiol Heart Circ Physiol.

[33] Creemers EE, Sutherland LB, Oh J, Barbosa AC, Olson EN (2006). Coactivation of MEF2 by the SAP domain proteins myocardin and MASTR. Mol Cell.

[34] van der Veer EP, de Bruin RG, Kraaijeveld AO, de Vries MR, Bot I, Pera T, Segers FM, Trompet S, van Gils JM, Roeten MK, Beckers CM, van Santbrink PJ, Janssen A, van Solingen C, Swildens J, de Boer HC, Peters EA, Bijkerk R, Rousch M, Doop M, Kuiper J, Schalij MJ, van der Wal AC, Richard S, van Berkel TJ, Pickering JG, Hiemstra PS, Goumans MJ, Rabelink TJ, de Vries AA, Quax PH, Jukema JW, Biessen EA, van Zonneveld AJ (2013). Quaking, an RNA-binding protein, is a critical regulator of vascular smooth muscle cell phenotype. Circ Res.

[35] Nakagaki-Silva EE, Gooding C, Llorian M, Jacob AG, Richards F, Buckroyd A, Sinha S, Smith CW (2019). Identification of RBPMS as a mammalian smooth muscle master splicing regulator via proximity of its gene with super-enhancers. Elife.

[36] Shukla S, Fisher SA (2008). Tra2beta as a novel mediator of vascular smooth muscle diversification. Circ Res.

[37] Zhou Y, Fan J, Zhu H, Ji L, Fan W, Kapoor I, Wang Y, Wang Y, Zhu G, Wang J (2017). Aberrant splicing induced by dysregulated Rbfox2 produces enhanced function of CaV1.2 calcium channel and vascular myogenic tone in hypertension. Hypertension.

[38] Li Z, Takakura N, Oike Y, Imanaka T, Araki K, Suda T, Kaname T, Kondo T, Abe K, Yamamura K (2003). Defective smooth muscle development in qkI-deficient mice. Dev Growth Differ.

[39] Dunn OJ, Clark V (1969). Correlation coefficients measured on the same individuals. J Am Stat Assoc.

[40] Zhu B, Rippe C, Thi Hien T, Zeng J, Albinsson S, Stenkula KG, Uvelius B, Swar K (2017). Similar regulatory mechanisms of caveolins and cavins by myocardin family coactivators in arterial and bladder smooth muscle. PLoS ONE.

[41] Liu L, Rippe C, Hansson O, Kryvokhyzha D, Fisher S, Ekman M, Swärd K (2021). Regulation of the muscarinic M3 receptor by myocardin-related transcription factors. Front Physiol.

[42] Dobin A, Davis CA, Schlesinger F, Drenkow J, Zaleski C, Jha S, Batut P, Chaisson M, Gingeras TR (2013). STAR: ultrafast universal RNA-seq aligner. Bioinformatics.

[43] Liao Y, Smyth GK, Shi W (2014). featureCounts: an efficient general purpose program for assigning sequence reads to genomic features. Bioinformatics.

[44] Love MI, Huber W, Anders S (2014). Moderated estimation of fold change and dispersion for RNA-seq data with DESeq2. Genome Biol.

[45] Mehmood A, Laiho A, Venalainen MS, McGlinchey AJ, Wang N, Elo LL (2020). Systematic evaluation of differential splicing tools for RNA-seq studies. Brief Bioinform.

[46] Anders S, Reyes A, Huber W (2012). Detecting differential usage of exons from RNA-seq data. Genome Res.

[47] Liao Y, Wang J, Jaehnig EJ, Shi Z, Zhang B, WebGestalt (2019). gene set analysis toolkit with revamped UIs and APIs. Nucleic Acids Res.

[48] Shen S, Park JW, Lu ZX, Lin L, Henry MD, Wu YN, Zhou Q, Xing Y (2014). rMATS: robust and flexible detection of differential alternative splicing from replicate RNA-Seq data. Proc Natl Acad Sci U S A.

[49] Vitting-Seerup K, Sandelin A (2019). IsoformSwitchAnalyzeR: analysis of changes in genome-wide patterns of alternative splicing and its functional consequences. Bioinformatics.

[50] Love MI, Soneson C, Patro R (2018). Swimming downstream: statistical analysis of differential transcript usage following Salmon quantification. F1000Res.

[51] Gohr A, Irimia M (2019). Matt: Unix tools for alternative splicing analysis. Bioinformatics.

[52] Sward K, Krawczyk KK, Moren B, Zhu B, Matic L, Holmberg J, Hedin U, Uvelius B, Stenkula K, Rippe C (2019). Identification of the intermediate filament protein synemin/SYNM as a target of myocardin family coactivators. Am J Physiol Cell Physiol.

[53] Dahan D, Ekman M, Larsson-Callerfelt AK, Turczynska K, Boettger T, Braun T, Sward K, Albinsson S (2014). Induction of angiotensin-converting enzyme after miR-143/145 deletion is critical for impaired smooth muscle contractility. Am J Physiol Cell Physiol.

[54] Zhu B, Rippe C, Holmberg J, Zeng S, Perisic L, Albinsson S, Hedin U, Uvelius B, Sward K (2018). Nexilin/NEXN controls actin polymerization in smooth muscle and is regulated by myocardin family coactivators and YAP. Sci Rep.

[55] Rippe C, Moren B, Liu L, Stenkula KG, Mustaniemi J, Wennstrom M, Sward K (2021). NG2/CSPG4, CD146/MCAM and VAP1/AOC3 are regulated by myocardin-related transcription factors in smooth muscle cells. Sci Rep.

[56] Gerstberger S, Hafner M, Tuschl T (2014). A census of human RNA-binding proteins. Nat Rev Genet.

[57] Somlyo AP, Somlyo AV (1994). Signal transduction and regulation in smooth muscle. Nature.

[58] Chen M, Zhang W, Lu X, Hoggatt AM, Gunst SJ, Kassab GS, Tune JD, Herring BP (2013). Regulation of 130-kDa smooth muscle myosin light chain kinase expression by an intronic CArG element. J Biol Chem.

[59] Khromov AS, Wang H, Choudhury N, McDuffie M, Herring BP, Nakamoto R, Owens GK, Somlyo AP, Somlyo AV (2006). Smooth muscle of telokin-deficient mice exhibits increased sensitivity to Ca2+ and decreased cGMP-induced relaxation. Proc Natl Acad Sci U S A.

[60] Yin F, Herring BP (2005). GATA-6 can act as a positive or negative regulator of smooth muscle-specific gene expression. J Biol Chem.

[61] Yin F, Hoggatt AM, Zhou J, Herring BP (2006). 130-kDa smooth muscle myosin light chain kinase is transcribed from a CArG-dependent, internal promoter within the mouse mylk gene. Am J Physiol Cell Physiol.

[62] Kohama K, Ye LH, Hayakawa K, Okagaki T (1996). Myosin light chain kinase: an actin-binding protein that regulates an ATP-dependent interaction with myosin. Trends Pharmacol Sci.

[63] Shrine N, Guyatt AL, Erzurumluoglu AM, Jackson VE, Hobbs BD, Melbourne CA, Batini C, Fawcett KA, Song K, Sakornsakolpat P, Li X, Boxall R, Reeve NF, Obeidat M, Zhao JH, Wielscher M, Weiss S, Kentistou KA, Cook JP, Sun BB, Zhou J, Hui J, Karrasch S, Imboden M, Harris SE, Marten J, Enroth S, Kerr SM, Surakka I, Vitart V, Lehtimaki T, Allen RJ, Bakke PS, Beaty TH, Bleecker ER, Bosse Y, Brandsma CA, Chen Z, Crapo JD, Danesh J, DeMeo DL, Dudbridge F, Ewert R, Gieger C, Gulsvik A, Hansell AL, Hao K, Hoffman JD, Hokanson JE, Homuth G, Joshi PK, Joubert P, Langenberg C, Li X, Li L, Lin K, Lind L, Locantore N, Luan J, Mahajan A, Maranville JC, Murray A, Nickle DC, Packer R, Parker MM, Paynton ML, Porteous DJ, Prokopenko D, Qiao D, Rawal R, Runz H, Sayers I, Sin DD, Smith BH, Soler Artigas M, Sparrow D, Tal-Singer R, Timmers P, Van den Berge M, Whittaker JC, Woodruff PG, Yerges-Armstrong LM, Troyanskaya OG, Raitakari OT, Kahonen M, Polasek O, Gyllensten U, Rudan I, Deary IJ, Probst-Hensch NM, Schulz H, James AL, Wilson JF, Stubbe B, Zeggini E, Jarvelin MR, Wareham N, Silverman EK, Hayward C, Morris AP (2019). New genetic signals for lung function highlight pathways and chronic obstructive pulmonary disease associations across multiple ancestries. Nat Genet.

[64] Sakaue S, Kanai M, Tanigawa Y, Karjalainen J, Kurki M, Koshiba S, Narita A, Konuma T, Yamamoto K, Akiyama M, Ishigaki K, Suzuki A, Suzuki K, Obara W, Yamaji K, Takahashi K, Asai S, Takahashi Y, Suzuki T, Shinozaki N, Yamaguchi H, Minami S, Murayama S, Yoshimori K, Nagayama S, Obata D, Higashiyama M, Masumoto A, Koretsune Y, FinnGen K, Ito C, Terao T, Yamauchi I, Komuro T, Kadowaki G, Tamiya M, Yamamoto Y, Nakamura M, Kubo Y, Murakami K, Yamamoto Y, Kamatani A, Palotie MA, Rivas MJ, Daly KM, Okada Y (2021). A cross-population atlas of genetic associations for 220 human phenotypes. Nat Genet.

[65] Zheng X, Reho JJ, Wirth B, Fisher SA (2015). TRA2beta controls Mypt1 exon 24 splicing in the developmental maturation of mouse mesenteric artery smooth muscle. Am J Physiol Cell Physiol.

[66] Dippold RP, Fisher SA (2014). Myosin phosphatase isoforms as determinants of smooth muscle contractile function and calcium sensitivity of force production. Microcirculation.

[67] Huttlin EL, Ting L, Bruckner RJ, Gebreab F, Gygi MP, Szpyt J, Tam S, Zarraga G, Colby G, Baltier K, Dong R, Guarani V, Vaites LP, Ordureau A, Rad R, Erickson BK, Wuhr M, Chick J, Zhai B, Kolippakkam D, Mintseris J, Obar RA, Harris T, Artavanis-Tsakonas S, Sowa ME, De Camilli P, Paulo JA, Harper JW, Gygi SP (2015). The BioPlex network: a systematic exploration of the human interactome. Cell.

[68] Ackers-Johnson M, Talasila A, Sage AP, Long X, Bot I, Morrell NW, Bennett MR, Miano JM, Sinha S (2015). Myocardin regulates vascular smooth muscle cell inflammatory activation and disease. Arterioscler Thromb Vasc Biol.

[69] Minami T, Kuwahara K, Nakagawa Y, Takaoka M, Kinoshita H, Nakao K, Kuwabara Y, Yamada Y, Yamada C, Shibata J, Usami S, Yasuno S, Nishikimi T, Ueshima K, Sata M, Nakano H, Seno T, Kawahito Y, Sobue K, Kimura A, Nagai R, Nakao K (2012). Reciprocal expression of MRTF-A and myocardin is crucial for pathological vascular remodelling in mice. EMBO J.

[70] Xu Y, Liang C, Luo Y, Zhang T (2021). MBNL1 regulates isoproterenol-induced myocardial remodelling in vitro and in vivo. J Cell Mol Med.

[71] Gan P, Wang Z, Morales MG, Zhang Y, Bassel-Duby R, Liu N, Olson EN (2022). RBPMS is an RNA-binding protein that mediates cardiomyocyte binucleation and cardiovascular development. Dev Cell.

[72] He WQ, Peng YJ, Zhang WC, Lv N, Tang J, Chen C, Zhang CH, Gao S, Chen HQ, Zhi G, Feil R, Kamm KE, Stull JT, Gao X, Zhu MS (2008). Myosin light chain kinase is central to smooth muscle contraction and required for gastrointestinal motility in mice. Gastroenterology.

[73] Backman L (2015). Calcium affinity of human alpha-actinin 1. PeerJ.

[74] Guo H, Huang R, Semba S, Kordowska J, Huh YH, Khalina-Stackpole Y, Mabuchi K, Kitazawa T, Wang CL (2013). Ablation of smooth muscle caldesmon affects the relaxation kinetics of arterial muscle. Pflugers Arch.

[75] Kanoldt V, Kluger C, Barz C, Schweizer AL, Ramanujam D, Windgasse L, Engelhardt S, Chrostek-Grashoff A, Grashoff C (2020). Metavinculin modulates force transduction in cell adhesion sites. Nat Commun.

[76] Surks HK, Mochizuki N, Kasai Y, Georgescu SP, Tang KM, Ito M, Lincoln TM, Mendelsohn ME (1999). Regulation of myosin phosphatase by a specific interaction with cGMP- dependent protein kinase Ialpha. Science.

[77] Imamura M, Long X, Nanda V, Miano JM (2010). Expression and functional activity of four myocardin isoforms. Gene.

