# Supplementary figures and images for: Myocardin regulates exon usage in smooth muscle cells through induction of splicing regulatory factors
Source: Cell Mol Life Sci. 2022 Aug 1;79(8):459. doi: 10.1007/s00018-022-04497-7 (PMC9343278; doi:10.1007/s00018-022-04497-7)

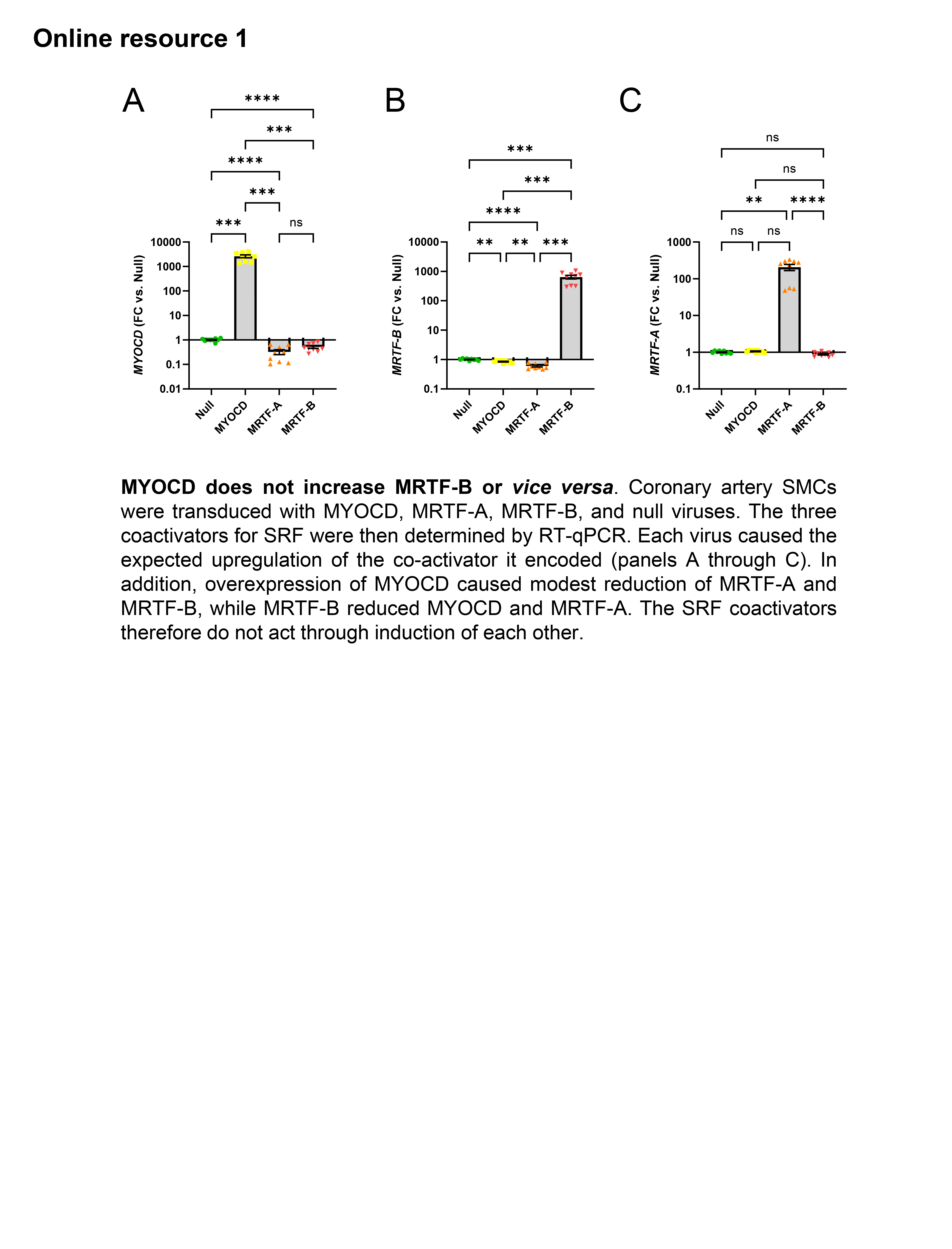

Supplement: Supplementary file 1 — Supplementary file1 (TIF 3580 KB) [file 18_2022_4497_MOESM1_ESM.tif]

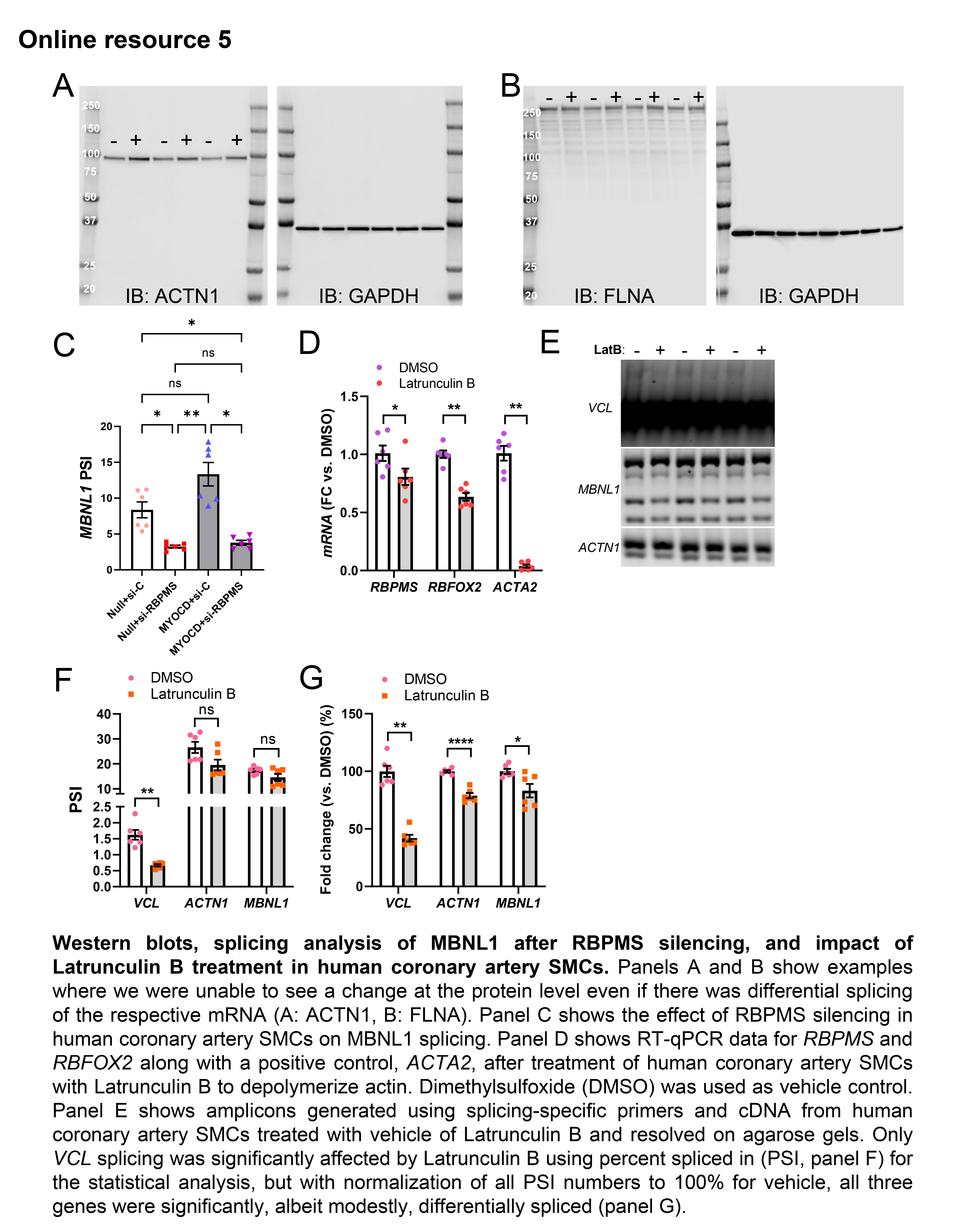

Supplement: Supplementary file 5 — Supplementary file5 (TIF 9343 KB) [file 18_2022_4497_MOESM5_ESM.tif]

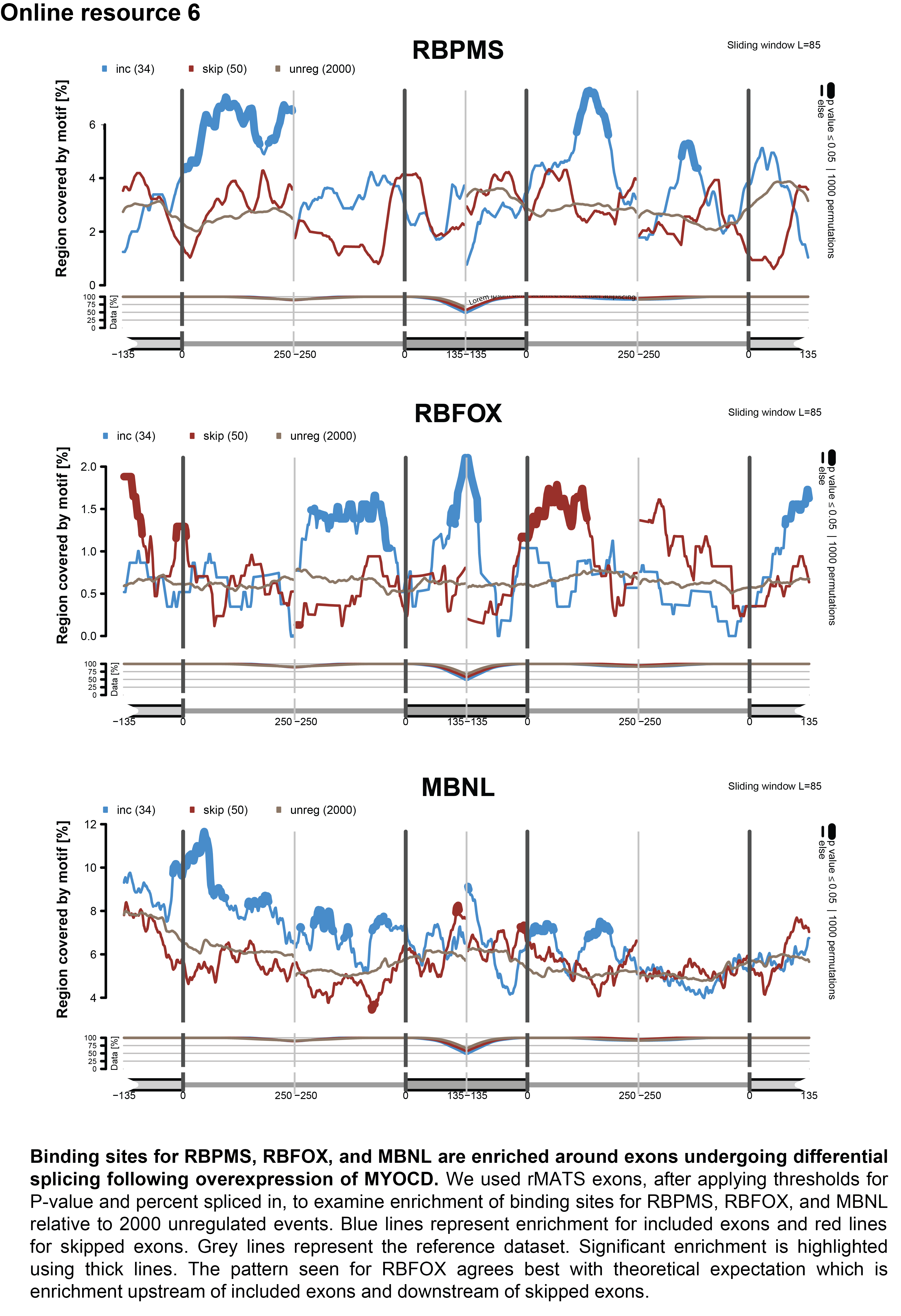

Supplement: Supplementary file 6 — Supplementary file6 (TIF 61868 KB) [file 18_2022_4497_MOESM6_ESM.tif]
